# Research progress on the N protein of porcine reproductive and respiratory syndrome virus

**DOI:** 10.3389/fmicb.2024.1391697

**Published:** 2024-04-29

**Authors:** Yajie Zheng, Gan Li, Qin Luo, Huiyang Sha, Hang Zhang, Ruining Wang, Weili Kong, Jiedan Liao, Mengmeng Zhao

**Affiliations:** ^1^School of Life Science and Engineering, Foshan University, Foshan, China; ^2^College of Veterinary Medicine, Henan University of Animal Husbandry and Economy, Zhengzhou, China; ^3^Gladstone Institutes of Virology and Immunology, University of California, San Francisco, San Francisco, CA, United States

**Keywords:** porcine reproductive and respiratory syndrome virus N protein, genetic evolution, protein interactions, detection techniques, vaccine development

## Abstract

Porcine reproductive and respiratory syndrome (PRRS) is a highly contagious disease caused by the porcine reproductive and respiratory syndrome virus (PRRSV). PRRSV exhibits genetic diversity and complexity in terms of immune responses, posing challenges for eradication. The nucleocapsid (N) protein of PRRSV, an alkaline phosphoprotein, is important for various biological functions. This review summarizes the structural characteristics, genetic evolution, impact on PRRSV replication and virulence, interactions between viral and host proteins, modulation of host immunity, detection techniques targeting the N protein, and progress in vaccine development. The discussion provides a theoretical foundation for understanding the pathogenic mechanisms underlying PRRSV virulence, developing diagnostic techniques, and designing effective vaccines.

## 1 Introduction

Porcine reproductive and respiratory syndrome (PRRS) is a viral infectious disease adversely affecting the global pig farming industry with significant economic harm (Cao et al., 2014; Lunney et al., 2016). This disease causes severe damage in pigs at various stages of growth, with clinical symptoms primarily manifesting as respiratory distress in piglets, reproductive failure, and congenital infections in pregnant animals (Chand et al., 2012). Financial losses in the United States caused by porcine reproductive and respiratory syndrome virus (PRRSV) amounted to $664 million in 2013 alone (Holtkamp et al., 2005; Lalonde et al., 2020). According to economic estimates in Germany, the average farm profit loss due to PRRSV was −19.1%, reaching up to −41% in the worst-case scenario in 2021 (Renken et al., 2021). As a global swine pathogen, PRRSV causes significant economic losses and merits sustained and widespread attention (Du et al., 2017).

PRRSV was first identified in the Netherlands in 1991 (Wensvoort et al., 1991). The virus was subsequently discovered in the United States in 1992 (Benfield et al., 1992; Collins et al., 1992). China documented an inaugural instance of PRRS in 1995, and an outbreak of highly pathogenic PRRS (HP-PRRS) occurred in 2006. The NADC30 and NADC34 variants have become the predominant circulating strains of PRRSV in China in recent years (Li et al., 2016; Guo et al., 2018; Bao and Li, 2021). PRRSV strains exhibit remarkable genetic and antigenic heterogeneity and frequently undergo recombination events, resulting in the emergence of a plethora of novel strains (Wang L. et al., 2017; Yu et al., 2020, 2022; Sun et al., 2022). The complexity of PRRSV genetic variability and recombination increases challenges to epidemiology, prevention, and control of PRRSV (Zhao et al., 2015; Zhou et al., 2015; Wang et al., 2018).

**Figure 1:**
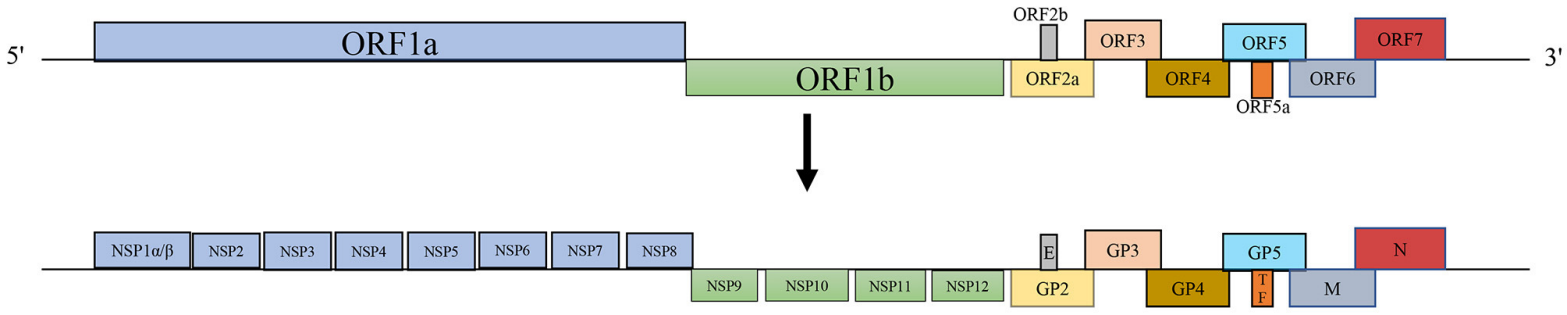
PRRSV genome structures.

PRRSV, a positive-sense RNA virus belonging to the family *Arteriviridae* within the order *Nidovirales*, has a genome length of approximately 15 kb with a 5′ cap and a 3′ poly-A tail (Balka et al., 2015; Lalonde et al., 2020). Over the years, increasing genetic differences have led to the classification of the virus into two distinct species: *Betaarterivirus suid-1* (PRRSV-1) and *Betaarterivirus suid-2* (PRRSV-2) (Brinton et al., 2018). PRRSV contains at least 11 open reading frames (ORFs), including ORF1a, ORF1b, ORF2a, ORF2b, ORFs3-7, ORF5a, and ORF2TF (Fang et al., 2012). ORF1a and ORF1b occupy approximately 75% of the viral genome at the 5′ end and encode two large polyprotein precursors, pp1a and pp1ab, processed into at least 16 non-structural proteins (Nsps), including Nsp1α, Nsp1β, Nsp2-6, Nsp2TF, Nsp2N, Nsp7α, Nsp7β, and Nsp8-12 (Fang et al., 2012; Li et al., 2019). ORF2a, ORF2b, ORFs3-7, and ORF5a are located at the 3′ end of the genome and encode glycoprotein (GP)2, envelope (E), GP3, GP4, GP5, ORF5a, membrane (M), and nucleocapsid (N) proteins (Fang and Snijder, 2010) (Figure 1). The largest difference in the sequence between PRRSV and other *Arteriviruses* lies in the N-protein domain (Meng, 2000).

The N protein is an immunogenic structural protein implicated in immune evasion (Music and Gagnon, 2010). This comprehensive review provides insights into the structural characteristics of the N protein, its genetic evolution, effects on PRRSV replication and virulence, interactions with other PRRSV and host proteins, modulation of host immunity, clinical detection and diagnosis, and applications of these findings in vaccine development. The information presented in this review lays the groundwork for enhancing our understanding of the pathogenic mechanisms associated with PRRSV and facilitating the development of commercially viable vaccines and diagnostic tools that specifically target the N protein of PRRSV.

## 2 Characteristics of the N protein

The alkaline phosphatase protein, known as the N protein, is versatile in various virus replication and pathogenesis mechanisms. This protein is encoded by the *ORF7* gene and has a molecular weight of approximately 15 kDa (Wootton et al., 2002). The N protein is a conserved peptide in PRRSV and is abundantly expressed owing to its discontinuous transcription mechanism, accounting for 40% of the total viral protein (Mardassi et al., 1994; Ke and Yoo, 2017). The amino acid residues at positions 105 (Y^105^) and 120 (I^120^) of the N protein are phosphorylation sites crucial for its functionality and involvement in viral replication (Chen Y. et al., 2018). The N protein has two domains: an RNA-binding domain at the N-terminus and a dimerization domain at the C-terminus. The N-terminal region, consisting of residues 1–57, is mainly disordered and contains several positively charged residues (Yoo et al., 2003). This region, rich in basic amino acid residues, promotes interactions between the N protein and RNA genome. The C-terminal region (residues 58–123) adopts a crystal structure comprising four antiparallel β-strands arranged in a dimeric form, surrounded by α-helices on its top and sides (Dokland et al., 2004; Dokland, 2010). This structure is crucial for maintaining the antigenicity of the N protein, the primary antigenic protein in PRRSV (Rowland and Yoo, 2003; Forsberg, 2005). The last 11 residues at the C-terminus of the N protein are essential for maintaining the structural integrity of the tertiary conformation (Wootton et al., 2001; Yoo and Wootton, 2001; Lee et al., 2005).

The N protein is distributed in both the cytoplasm and nucleus and regulates host cell processes within the nucleus (Rowland et al., 1999). Rowland et al. (2003) identified two potential nuclear localization signals (NLS) in the N protein of PRRSV-2 isolates. NLS-1 and NLS-2 are at amino acid positions 10–13 and 41–42, respectively. The transport of the N protein involves the participation of a single NLS, enabling its translocation from the cytoplasm to the nucleus. NLS-2 targets the N protein to the nucleolus. There is an NLS between 41–47 aa of the N protein, and these residues serve as highly conserved determinants (Wootton et al., 1998). You et al. (2008) found that the N protein exhibited a relatively higher distribution within the nucleus than in the cytoplasm, with a faster nuclear import rate than export.

The PRRSV N protein is involved in viral nucleocapsid formation, encapsulating the viral genome and enabling virus assembly. The N protein can form homodimers, indicating that it can interact with other N protein molecules to form a dimeric structure (Jourdan et al., 2011; Snijder et al., 2013). The N protein assembles into a spherical structure with a diameter of 20–30 nm as a dimer. Three conserved cysteine residues within the protein form disulfide bonds that stabilize the spherical structure (Doan and Dokland, 2003; Wootton and Yoo, 2003; Lee et al., 2004). This homodimeric structure is essential for binding to the viral genome and facilitating viral particle assembly.

The RNA-binding domain located at the N-terminus and the dimerization domain at the C-terminus of the N protein are vital for viral replication, maintaining N protein antigenicity, and maintaining protein tertiary conformation. Additionally, two potential NLS of the N protein facilitate its transport from the cytoplasm to the nucleus and then to the nucleolus. The N protein shows a dynamic distribution between the cytoplasm and the nucleolus, with quicker nuclear entry compared to nuclear export. Moreover, the N protein is crucial for the formation of viral nucleocapsids, and its homodimeric structure is necessary for binding to RNA genomes and assembling viral particles.

## 3 Genetic evolution of the N protein

**Figure 2:**
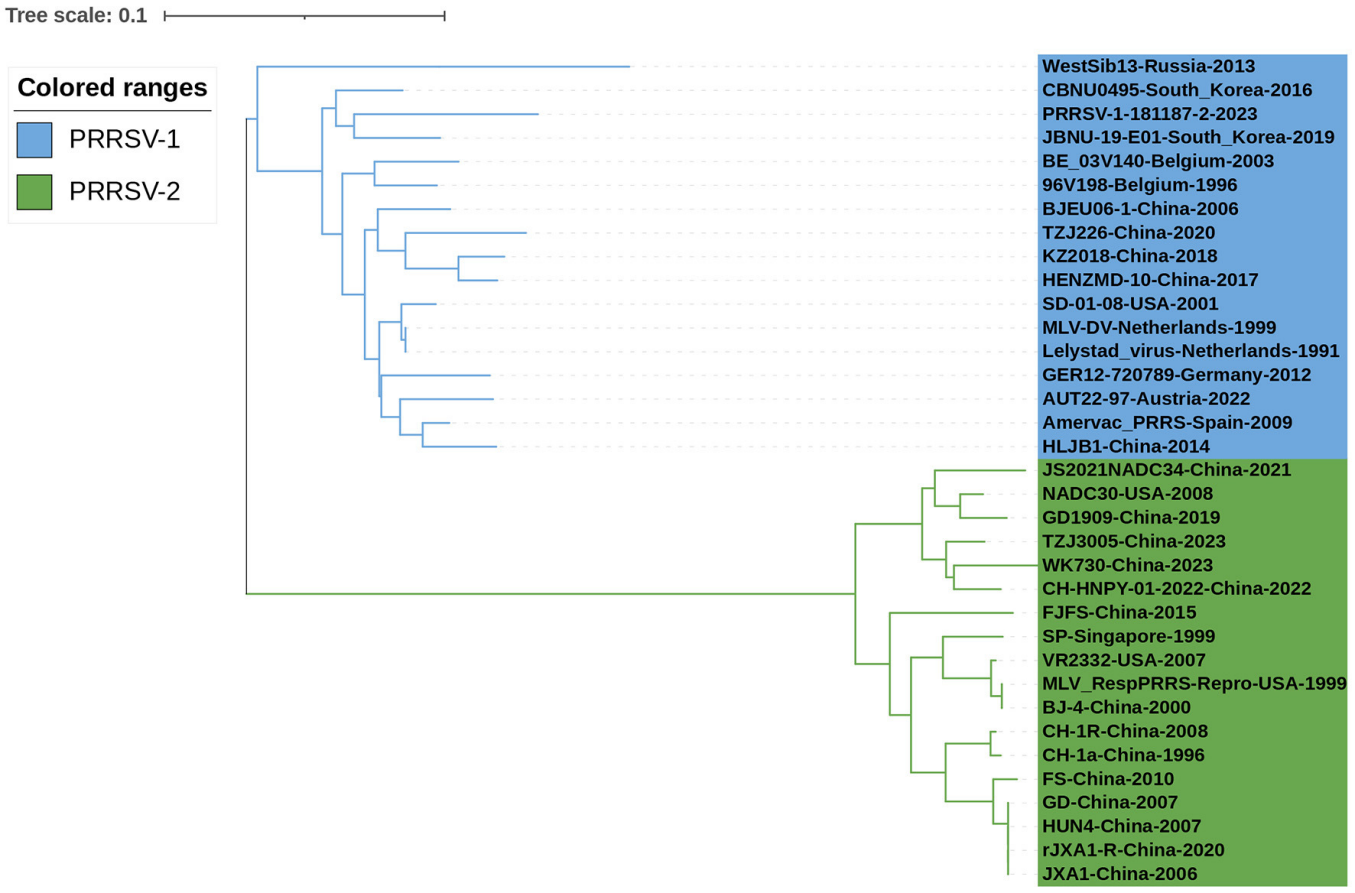
Phylogenetic analysis of the N gene. First, Clustal W alignment was conducted using the MegAlign feature in DNAStar software (version 7.0). Subsequently, the neighbor-joining method with 1000 bootstrap replicates was performed using the MEGA software (version 7.0). The resulting tree was visualized and annotated using the online “The Interactive Tree of Life” (https://itol.embl.de, accessed on 17 January 2024) software. PRRSV-1 and PRRSV-2 strains are represented in blue and green, respectively.

Thirty-six PRRSV N sequences were selected from the NCBI nucleotide database to analyze the genetic evolution characteristics of the N protein (Table 1). The selected strains included isolates from different years, ranging from 1991 to 2023, vaccine strains, and widely referenced representative strains. Phylogenetic analysis was conducted on the selected 36 PRRSV N protein sequences (Figure 2). Among the PRRSV-1 strains, WestSib13-Russia-2013 and PRRSV-1-181187-2-2023 exhibited a greater genetic distance, while MLV-DV-Netherlands-1999 and Lelystad virus-Netherlands-1991 showed a closer genetic relationship. Among the PRRSV-2 strains, JS2021NADC34-China-2021 and wK730-China-2023 showed greater genetic distances, whereas NADC30-USA-2008 and TZJ3005-China-2023 exhibited a closer genetic relationship. In 1995, Meng et al. (1995) conducted an amino acid conservation study on the M and N genes of PRRSV isolates from the United States and Canada, leading to the classification of PRRSV isolates into two groups. Based on their evolutionary relationships, Figure 2 categorizes the PRRSV strains into two major branches, designated type 1 and type 2.

**Table 1:** Information about the 36 selected PRRSV strains.

**Year**	**Area**	**Strain**	**Genbank accession number**	**Genotype**
1991	Netherlands	Lelystad virus	M96262	PRRSV-1
1996	Belgium	96V198	MK876228	PRRSV-1
1999	Netherlands	MLV-DV	KJ127878	PRRSV-1
2001	USA	SD-01-08	DQ489311	PRRSV-1
2003	Belgium	BE_03V140	MW053394	PRRSV-1
2006	China	BJEU06-1	GU047344	PRRSV-1
2008	Belarus	lena	JF802085	PRRSV-1
2009	Spain	Amervac PRRS	GU067771	PRRSV-1
2012	Germany	GER12-720789	OP529852	PRRSV-1
2013	Russia	WestSib13	KX668221	PRRSV-1
2014	China	HLJB1	KT224385	PRRSV-1
2016	South Korea	CBNU0495	MZ287327	PRRSV-1
2017	China	HENZMD-10	KY363382	PRRSV-1
2018	China	KZ2018	MN550991	PRRSV-1
2019	South Korea	JBNU-19-E01	MW847781	PRRSV-1
2020	China	TZJ226	OP566682	PRRSV-1
2022	Austria	AUT22-97	OP627116	PRRSV-1
2023	China	PRRSV-1-181187-2-2023	OQ856755.1	PRRSV-1
1996	China	CH-1a	AY032626	PRRSV-2
1999	USA	MLV RespPRRS-Repro	AF159149	PRRSV-2
1999	Singapore	SP	AF184212.1	PRRSV-2
2000	China	BJ-4	AF331831	PRRSV-2
2006	China	JXA1	EF112445	PRRSV-2
2007	USA	VR2332	EF536003	PRRSV-2
2007	China	HUN4	EF635006	PRRSV-2
2007	China	GD	EU109503	PRRSV-2
2008	USA	NADC30	JN654459	PRRSV-2
2008	China	CH-1R	EU807840.1	PRRSV-2
2010	China	FS	JF796180.1	PRRSV-2
2015	China	FJFS	KP998476	PRRSV-2
2019	China	GD1909	MT165636	PRRSV-2
2020	China	rJXA1-R	MT163314.1	PRRSV-2
2021	China	JS2021NADC34	MZ820388	PRRSV-2
2022	China	CH-HNPY-01-2022	OP716076.1	PRRSV-2
2023	China	WK730	OR826314	PRRSV-2
2023	China	TZJ3005	OR826313	PRRSV-2

**Figure 3:**
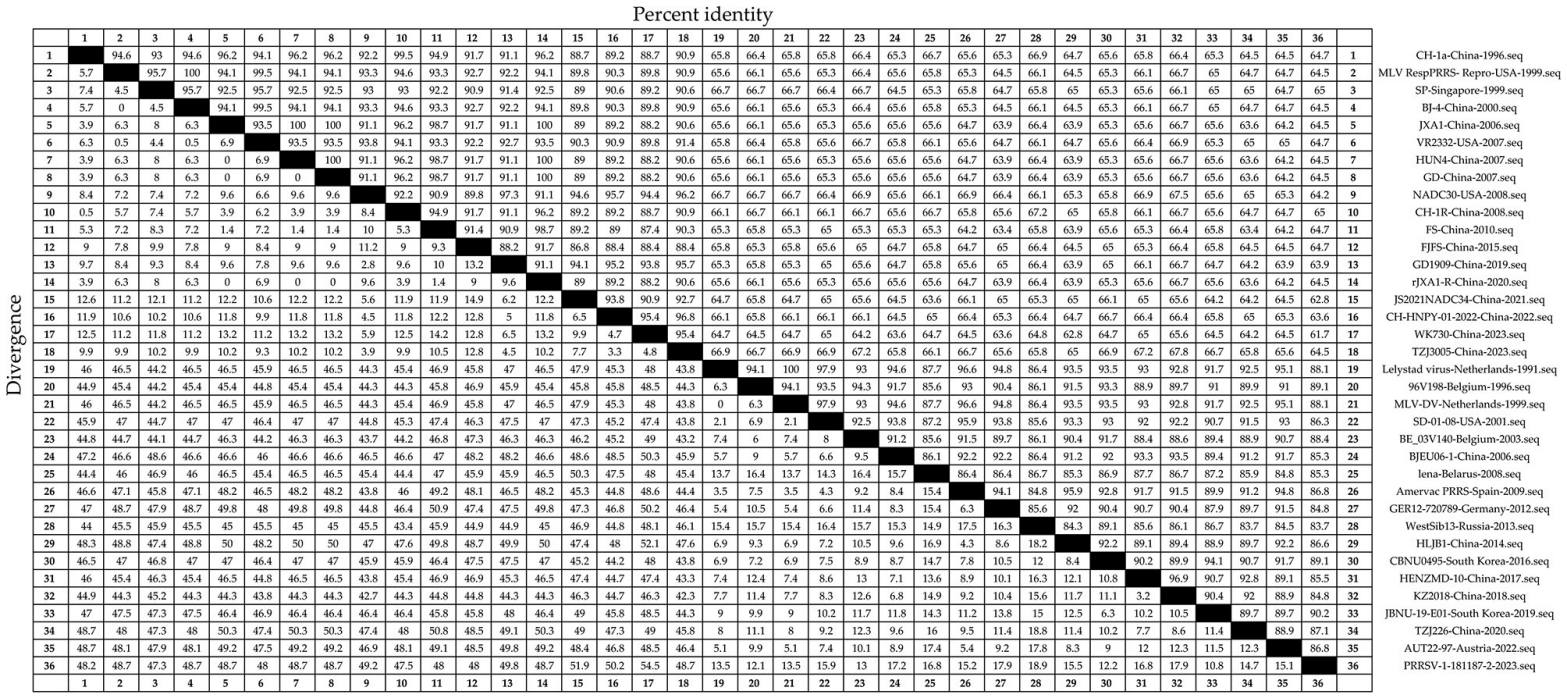
Thirty-six representative PRRSV strains were selected (Table 1). The N sequences were obtained and analyzed for nucleotide homology. The Clustal W algorithm in DNAStar software (version 7.0, Madison, WI, USA) was used to analyze the nucleotide homology of the N sequences.

Nucleotide homology between PRRSV-1 N and PRRSV-2 N was 61.7%−100% (Figure 3). The strains with 61.7% nucleotide sequence homology were WK730-China-2023 and PRRSV-1-181187-2-2023. Strains with 100% nucleotide homology were Lelystad virus Netherlands 1991, MLV-DV Netherlands 1999, JXA1 China 2006, and HUN4 China 2007. Nucleotide homology within the PRRSV-2 strain was 61.7%−100%. The same applied to strains with a nucleotide homology of 61.7%. HUN4 China-2007 and GD China-2007 showed 100% homology. Nucleotide homology between the PRRSV-1 strains ranged from 83.7% to 100%. The strains with 83.7% nucleotide homology were WestSib13-Russia-2013 and PRRSV-1-181187-2-2023. Lelystad virus Netherlands 1991 and MLV-DV Netherlands 1999 strains showed 100% homology. Therefore, the N sequence has high nucleotide homology and conservation.

**Figure 4:**
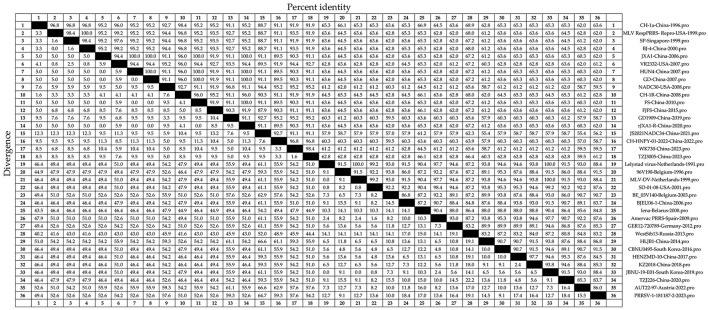
Thirty-six representative PRRSV strains (Table 1), including recently circulating strains, were selected. The N amino acid sequence homology was analyzed using DNAStar software (version 7.0, Madison, WI, USA) with the Clustal W algorithm in the MegAlign tool.

Amino acid homology analysis was conducted on the N sequences of the 36 strains of PRRSV (Figure 4). The amino acid homology between PRRSV-1 and PRRSV-2 was 55.4%−100%. JS2021NADC34-China-2021 and HLJB1-China-2014 strains showed 55.4% amino acid homology. SP-Singapore-1999 and BJ-4-China-2000, among others, showed 100% amino acid homology. The amino acid homology between the PRRSV-2 strains ranged from 55.4% to 100%, consistent with the amino acid homology between PRRSV-1 and PRRSV-2. Among the PRRSV-1 strains, amino acid homology ranged from 80.8% to 100%. Strains with 80.8% amino acid homology included WestSib13-Russia-2013 and TZJ226-China-2020. Strains with 100% amino acid homology included Lelystad virus-Netherlands-1991 and MLV-DV-Netherlands-1999. A relatively high conservation of N sequences at the amino acid level was inferred.

**Figure 5:**
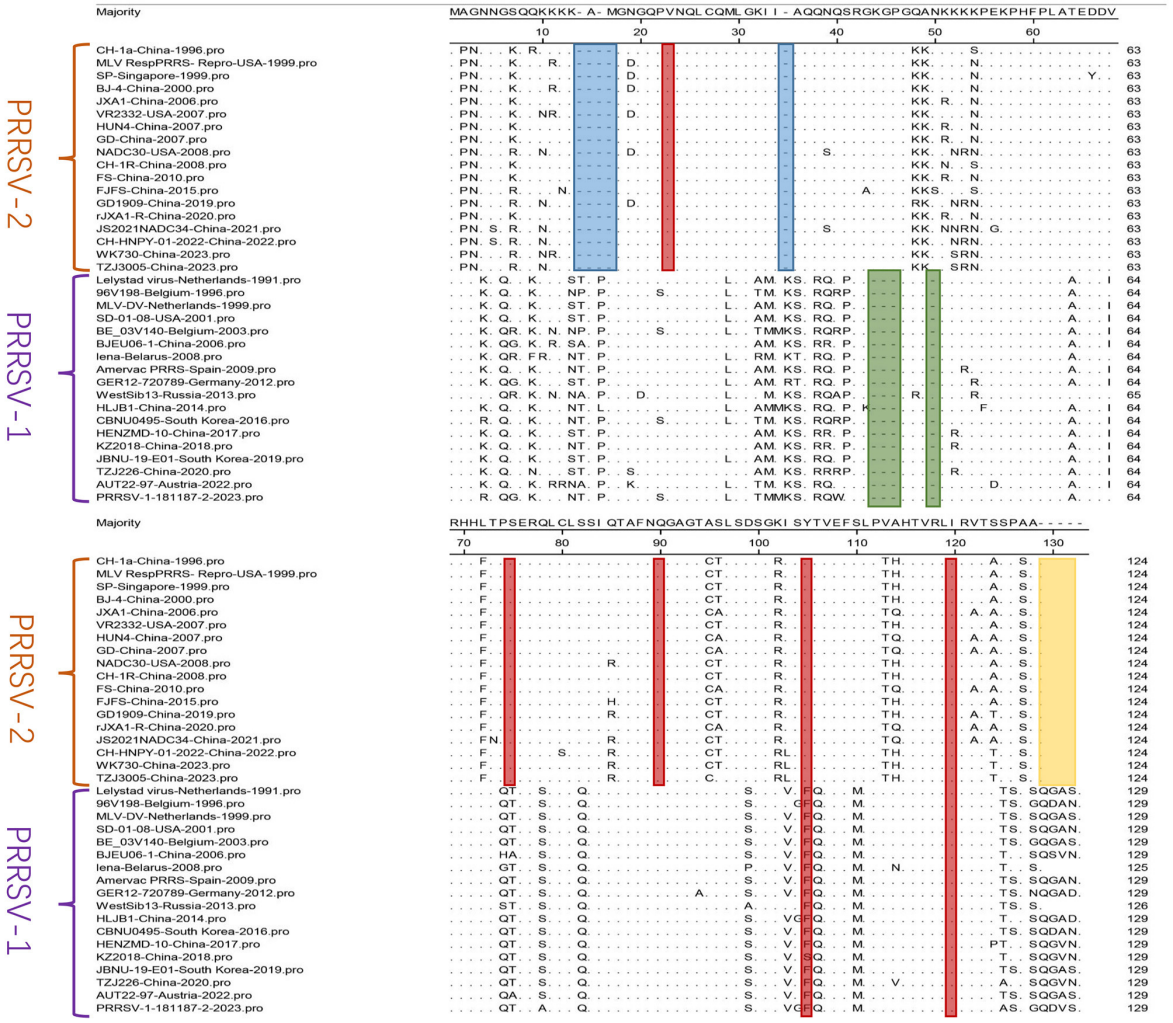
A comparison was performed for N sequences. The MegAlign sequence alignment editor in DNAStar software (version 7.0, Madison, WI) was used to analyze the amino acid sequences of N. The regions with multiple amino acid deletions are represented in blue, green, and yellow, and red indicates conserved amino acid positions.

Amino acid mutation analysis was performed on the N sequences of the 36 selected PRRSV strains (Figure 5). The results revealed amino acid deletions in PRRSV. In PRRSV-1, deletions were at positions 14–17 aa and 35, whereas in PRRSV-2, deletions were at positions 44–46 aa and 50. Amino acid residues at positions 105 and 120 of the N protein are critical phosphorylation sites for the functionality and viral replication capacity (Chen Y. et al., 2018). The PRRSV-2 N protein is highly conserved at positions 90, 75, and 23 aa, which are responsible for crucial functions such as N-N interactions and the formation of disulfide bonds (Lee and Yoo, 2005). The mutation analysis validated the above conclusions, as indicated by the red markings in Figure 5, showing conserved amino acid positions within PRRSV-1 and PRRSV-2. The N proteins of PRRSV-1 and PRRSV-2 comprise 123 and 128 amino acids, respectively (Mardassi et al., 1995; Meulenberg et al., 1995). The yellow portion in Figure 5 indicates amino acid deletions in PRRSV-2 compared to those in PRRSV-1. Although multiple amino acid mutations exist in PRRSV, significant differences in the amino acid profiles of the two genotypes exist. Overall, there are relatively few amino acid mutations and a high level of conservation within the internal regions of both PRRSV-1 and PRRSV-2. Gall et al. (1998) demonstrated the minimal effect of the *in vivo* passage of PRRSV-1 on the ORF7 sequence. Only a few detectable amino acid substitutions were observed in ORF7, confirming its low variability. The sequence alignment results obtained by Zhou et al. (2006) revealed the high conservation of antigenic epitopes in the N protein among PRRSV strains.

Analysis of N sequences from 36 PRRSV strains identified two major branches, PRRSV-1 and PRRSV-2, through phylogenetic tree analysis. Homology analysis showed nucleotide homology ranging from 61.7%−100% and amino acid homology ranging from 55.4%−100% between PRRSV-1 and PRRSV-2. Amino acid mutation analysis of the N sequence indicated a low occurrence of mutations within PRRSV-1 and PRRSV-2, emphasizing their internal conservation. These results underscore the high conservation level in the N sequence, emphasizing the importance of timely monitoring genetic evolution in the N protein for effective PRRS prevention and control.

## 4 Function of the N protein in viral replication

The N protein serves as a phosphorylated protein. Mutations in the phosphorylation sites may affect the replication capacity of PRRSV within cells (Wootton et al., 2002; Chen L. et al., 2018). The N protein can interact with the 3′ end of the viral genome to regulate the synthesis of viral RNA and affect viral replication (Fahad and Kapil, 2001). Disulfide bond formation and the NLS of the N protein are crucial for viral replication (Wootton and Yoo, 2003; Lee and Yoo, 2005; Lee et al., 2005; Pei et al., 2008).

Poly (ADP-ribose) polymerase-1 (PARP-1) is a cellular factor that adds ADP-ribose moieties to proteins. Liu L. et al. (2014) demonstrated PARP-1′s involvement in viral replication. PARP-1 interacts with the N protein and affects PRRSV replication (Zhao et al., 2019). During PRRSV infection, the N protein interacts with the RNA helicase DExD/H-box helicase 9 (DHX9) and affects viral replication (Liu et al., 2016). Wang C. et al. (2017) demonstrated the interaction of the N protein with the unique SUMO E2 conjugating enzyme Ubc9 and their co-localization in the cytoplasm and nucleus. The SUMOylation characteristics of the N protein were shown. Ubc9 inhibited viral replication through its interaction with the N protein. MicroRNAs (miRNAs) affect viral replication by binding to mRNAs (Krol et al., 2010). An et al. (2020) found that miR-10a-5p overexpression decreased the level of the N protein and indirectly suppressed PRRSV replication. The PRRSV N protein promotes PRRSV proliferation by activating CCAAT/Enhancer Binding Protein β to induce the expression of Transcription Factor Dp-2 (TFDP2). PRRSV can utilize host proteins to regulate the cell cycle for its own benefit during infection (Zhu et al., 2021).

S100A9 belongs to the S100 protein family and has a damage-associated molecular pattern (DAMP). It inhibits PRRSV replication in a Ca^2+^-dependent manner. Song et al. (2019) showed that the interaction between residues 36–37 aa of the N protein and amino acid residue 78 of S100A9 enabled their co-localization in the cytoplasm. This interaction restricted PRRSV proliferation. S100A9 may restrict PRRSV proliferation by interacting with the viral N protein. Specific protein 1 (SP1) is a transcription factor regulating various biological processes (Vizcaíno et al., 2015). Chen J. et al. (2017) demonstrated that the N protein enhanced the expression of miR-373 through SP1, thereby suppressing IFN-β expression and PRRSV replication. Zhao et al. (2018), through co-immunoprecipitation (Co-IP) and immunofluorescence co-localization, showed that Moloney leukemia virus 10 (MOV10) colocalized with the N protein in the cytoplasm. MOV10 causes cytoplasmic retention of the N protein and inhibits PRRSV replication by restricting its entry into the nucleus.

## 5 Effects of the N protein on viral virulence

Kwon et al. (2008) conducted an initial whole-genome scan using a model of reproductive failure in sows. Their results suggested that PRRSV's non-structural region (ORF1a and 1b) and structural region (ORF2-7) may contain virulence determinants, confirm the polygenic nature of PRRSV, and indicate that ORF7 is a potential determinant of virulence. This conclusion was supported by Wang et al. (2007). They constructed two chimeric PRRSV mutants by exchanging fragments of non-structural or structural genes. These gene fragments originated from attenuated vaccine strains and virulent strains. When pigs were inoculated with these two chimeric PRRSV mutants, they exhibited attenuated virulence. These findings unveil a strategy for the molecular generation of new attenuated vaccines.

Phosphorylation regulates the growth and virulence of various pathogens. However, whether phosphorylation of the N protein affects PRRSV virulence, remains unclear (Albataineh and Kadosh, 2016). Chen et al. (2019) demonstrated that certain mutations in the N protein reduced the replication capacity of PRRSV within cells. Experimental findings indicated that the mutant virus (A105-120) exhibited significantly lower pathogenicity than the parental virus strain (XH-GD). Taken together, phosphorylation of the N protein can affect virulence. No direct evidence suggests that the N protein affects PRRSV virulence. It is speculated that the N protein interacts with other structural or non-structural PRRSV proteins to affect viral virulence collectively. Additional studies are required to explore the effect of N proteins on viral virulence.

## 6 Interactions of the N protein with PRRSV proteins

PRRSV GP5 interacts with heparin sulfate glycosaminoglycan (HSGAG) and sialoadhesin/CD169 to facilitate viral entry (Veit et al., 2014; Shi et al., 2015). It interacts with the N protein to transport the viral RNA complex to specific sites (Montaner-Tarbes et al., 2019). Nuclear factor kappa B (NF-κB) is an inducible transcription factor. Lee and Kleiboeker (2005) found that PRRSV N protein and Nsp2 synergistically activated NF-κB. Lee and Yoo (2005) performed immunoprecipitation on cells co-expressing N and E proteins and demonstrated that the N protein did not co-precipitate with the PRRSV E protein under reducing or non-reducing conditions. A GST pull-down assay was conducted to confirm the specific interactions between N and N and between N and E, revealing the significance of non-covalent interactions between N and E proteins in PRRSV replication.

The above findings indicate that PRRSV GP5, Nsp2, and E can interact with the N protein. Understanding the interaction mechanisms between the N protein and other PRRSV proteins is crucial for developing drugs targeting the N protein.

## 7 N protein interacts with host proteins

**Figure 6:**
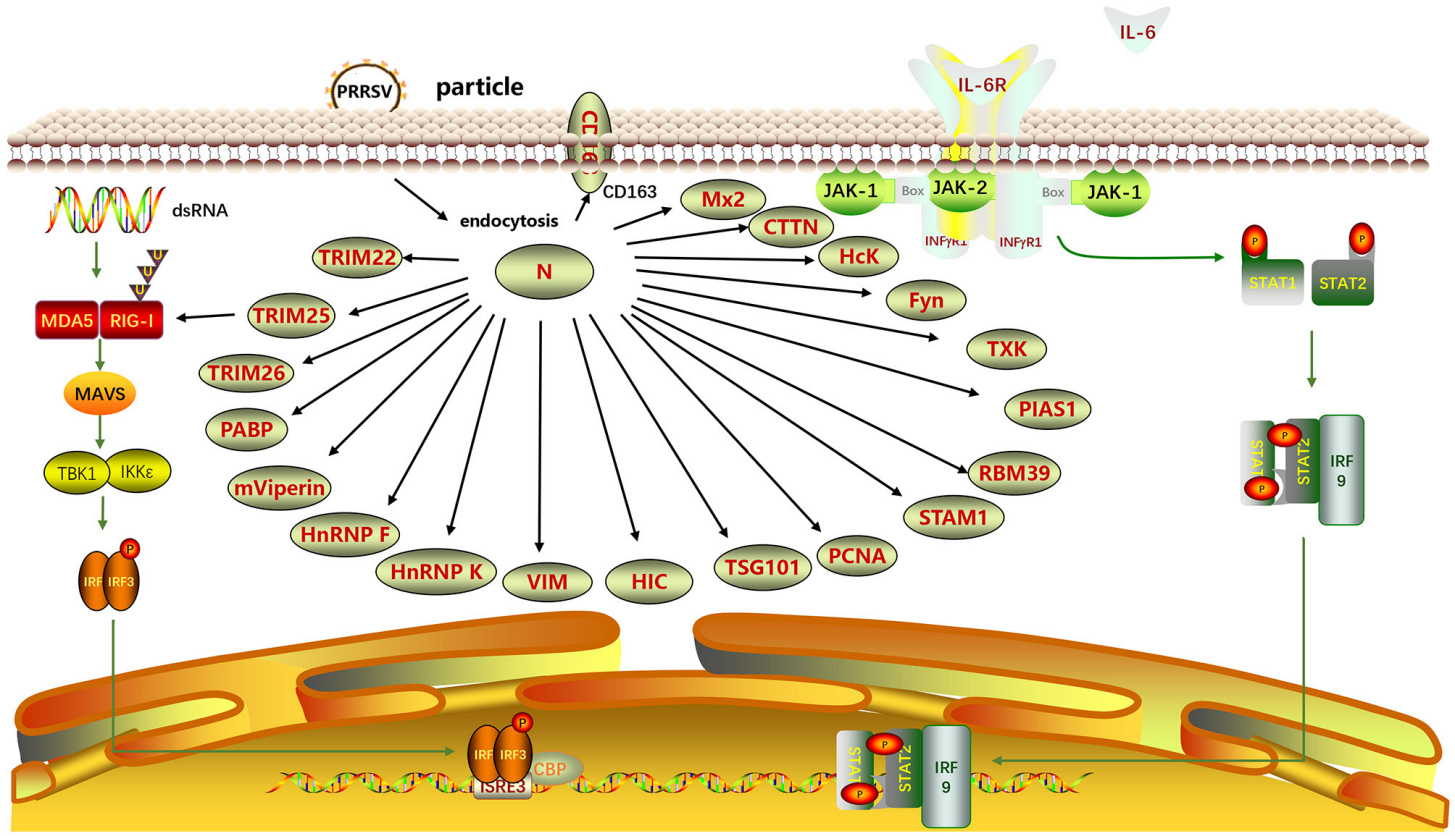
Interaction between N and host proteins. A protein-protein interaction network was generated using ScienceSlides software (version 2016). TRIM22, Tripartite motif-containing protein 22; TRIM25; TRIM26; PABP, Poly(A)-binding protein; mViperin, mouse Viperin; HnRNP F, Heterogeneous nuclear ribonucleoprotein F; HnRNP K; VIM, Vimentin; HIC, Hydrogenase-iron-only protein-like protein; TSG101, Tumor Susceptibility Gene 101; HcK, Hematopoietic cell kinase; PCNA, Proliferating Cell Nuclear Antigen; STAM1, Signal Transducing Adaptor Molecule 1; RBM39, RNA Binding Motif Protein 39; PIAS1, Protein Inhibitor of Activated STAT 1; TXK, Tec family kinase; Fyn, FYN proto-oncogene, Src family tyrosine kinase; CTTN, Cortactin; Mx2, Myxovirus resistance 2; CD163, Cluster of Differentiation 163.

The N protein performs diverse biological functions by interacting with multiple host proteins (Figure 6). Proteins of the tripartite motif (TRIM) family, including TRIM22, TRIM25, and TRIM26, interact with the PRRSV N protein. Jing et al. (2019) found that the SPRY domain of TRIM22 interacted with the NLS2 motif of the PRRSV N protein. TRIM22 did not alter the N protein concentration. However, Chen et al. (2022) showed that TRIM22 enhanced lysosomal pathway activation by inducing lysosomal degradation of N proteins through interaction with LC3 and inhibited PRRSV replication. Zhao et al. (2019) revealed that PRRSV N protein interfered with the interaction between TRIM25 and RIG-I through competitive interactions to mediate RIG-I ubiquitination and counteract viral activity. Zhao et al. (2022) revealed that TRIM26 could bind to the N protein through its C-terminal PRY/SPRY domain and induce N protein degradation, thereby inhibiting PRRSV replication.

Poly(A)-binding protein (PABP) enhances the mRNA translation rate. Through yeast two-hybrid (Y2H) screening for cellular interactors of the N protein, the interaction region between the N protein and PABP was identified as within the 52–69 aa region. This interaction affects the viral replication (Wang et al., 2012). Sagong and Lee (2010) discovered that monkey viperin (mviperin), a homolog of mouse viperin, interacts with N protein at different cellular locations within the cytoplasm. Overexpression of mviperin inhibits viral genome replication, highlighting the significance of in-depth investigations on the interaction between mviperin and the N protein (Fang et al., 2016). Heterogeneous nuclear ribonucleoprotein F (HnRNP F) and hnRNP K affect protein expression. Knocking down hnRNP F effectively blocks the synthesis of the viral N protein (Zhang A. et al., 2022). The overexpression of hnRNP K inhibits PRRSV replication (Jing et al., 2023). These findings highlight the potential involvement of hnRNP F and hnRNP K in modulating PRRSV replication, enhancing our comprehension of host-PRRSV interactions.

Vimentin (VIM) is a major type III intermediate filament protein. It stabilizes the cytoskeleton and maintains cell integrity (Goldman et al., 1996). VIM is an important component of the PRRSV receptor complex, contributing to intracellular replication and dissemination of PRRSV (Wang et al., 2011). VIM can bind to the PRRSV N protein, and anti-VIM antibodies can block PRRSV infection in MARC-145 cells. VIM forms complexes with PRRSV Nsp2 and N proteins, which may be crucial for viral attachment and replication (Song et al., 2016). The initiator of chromosome condensation (HIC) is a protein containing an I-MyoD family inhibitor (I-mfa) domain, a recently discovered cellular transcription factor, and a homolog of human HIC. Song et al. (2009) experimentally confirmed that the N protein and the HIC-p40 isoform colocalize in the cell nucleus, whereas they colocalize with HIC-p32 in the cytoplasm, a truncated N-terminal product of HIC-p40, through mammalian two-hybrid analysis and immunoprecipitation assay. The interaction between the viral N protein and cellular transcription factors suggests that the N protein modulates host cell gene expression during PRRSV infection. The CD163 SRCR5 domain in macrophages confers resistance to PRRSV infection. Yu et al. (2019) discovered that the CD163 SRCR5 domain colocalized with the N protein within cells during the early stages of infection and participated in the viral invasion of the host.

Tumor susceptibility gene 101 (TSG101) is crucial in PRRSV infection. TSG101, a subunit of the ESCRT-I complex, interacts with the N protein to promote the formation of PRRSV viral particles. TSG101 is a cellular protein that can facilitate PRRSV assembly (Zhang Q. et al., 2022). Proliferating cell nuclear antigen (PCNA) is involved in DNA repair. Wang et al. (2023) discovered that PCNA interacted with the replication-associated proteins Nsp9, Nsp12, and N of PRRSV through immunoprecipitation and immunofluorescence co-localization assays. Region III (41–72 aa) of the N protein interacts with the IDCL region (118–135 aa) of PCNA. Therefore, the cytoplasmic translocation of PCNA and its effect on PRRSV RNA synthesis may represent potential targets for controlling PRRSV infection.

The Src homology 3 (SH3)-binding motif (SH3BM) PxxPxxP (PxxP) is conserved in the PRRSV N protein. In the study by Kenney and Meng (2015), five host cell proteins were identified to interact with SH3, including hematopoietic cell kinase (Hck), Tec family kinase (TXK), cortactin (CTTN), Fyn Proto-Oncogene, Src Family Tyrosine Kinase (fyn), and signal transducing adaptor molecule (STAM) I. The binding of SH3 proteins to the PRRSV N protein depends on at least one PxxP motif. The interaction of STAMI and Hck with PRRSV N protein requires an unobstructed C-terminal structure. PRRSV promotes the expression of RNA-binding motif protein 39 (RBM39) within cells (Song et al., 2021). RBM39 interacts with PRRSV proteins, including Nsp4, M, and N (You et al., 2022). Ke et al. (2019) recently described the mechanisms of activation of NF-κB by PRRSV at the molecular level. PIAS1, a protein inhibitor of activated STAT1, interacts with N protein. The binding of the N protein to PIAS1 releases the p65 subunit of NF-κB, resulting in the activation of NF-κB. Myxovirus resistance 2 (Mx2) is a newly identified interferon-induced innate immune restriction factor that inhibits viral infections. Wang et al. (2016) reported the inhibitory effect of the porcine Mx2 protein on PRRSV replication, demonstrating the interaction between the Mx2 and N proteins of the virus leading to inhibition.

## 8 N protein's involvement in the host immune system

A PRRSV negatively regulates the host immune response, leading to persistent immunosuppression. PRRSV N protein is involved in this process by inhibiting the induction of type I IFN, IFN-β, and the phosphorylation of IRF3 (Sagong and Lee, 2011). The N protein has been demonstrated to suppress IFN-induced ISRE reporter expression and STAT2 elevation, while also hindering the nuclear translocation of STAT1. However, a comprehensive investigation into the specific functions and regulatory mechanisms of the N protein in the JAK/STAT signaling pathway requires the utilization of diverse experimental techniques and methods (Wang et al., 2013). PRRSV's inhibition of IFN by PRRSV is multifactorial. Several PRRSV proteins inhibit IFN, including Nsp1α, Nsp11, and N proteins (Yoo et al., 2010; Sun et al., 2012; Han and Yoo, 2014; Lunney et al., 2016).

PRRSV N protein is responsible for interleukin-10 (IL-10) production, and the non-covalent N-N domain is associated with this process (Hou et al., 2012; Liu et al., 2015; Yu et al., 2017). The N protein can trigger the expression of IL-10 in peripheral blood mononuclear cells (PBMCs) and monocyte-derived dendritic cells (MoDCs) (Wongyanin et al., 2012). Interleukin-15 (IL-15), a cytokine, promotes the production of various cells. The PRRSV N protein can induce the production of IL-15, which is mediated by multiple structural domains and activates NF-κB. The PRRSV N protein mediates NF-κB activation, leading to the induction of IL-15 production. This finding contributes to a deeper understanding of the mechanisms by which PRRSV infection induces IL-15 production (Fu et al., 2012).

The PRRSV N protein possesses multiple antigenic epitopes that form the basis for its involvement in the host immune system. Meulenberg et al. (1998) identified B-cell epitopes of the PRRSV-1 N protein at positions 2–12 and 25–30 aa using peptide scanning techniques. Plagemann et al. (2005) identified B cell epitopes of the PRRSV-2 N protein at positions 23–33, 31–50, and 43–56 aa using peptide scanning techniques. An et al. (2005) conducted a biopanning analysis using phage display and identified a B-cell epitope of the PRRSV II N protein at positions 78–87 aa. Identification of this epitope was based on monoclonal antibodies. Plagemann (2004) characterized epitopes at positions 24–32, 29–30, 31–39, 42–50, 50–60, and 54–92 aa, indicating that residues 23–92 aa may represent the dominant regions for B-cell epitopes. Wang et al. (2014) discovered that the B-cell epitope of the PRRSV I N protein was located at 1–15 aa. PRRSV infection increases the population of PRRSV-specific regulatory T lymphocytes (CD4+CD25+Foxp3+ Tregs) in infected pigs. Fan et al. (2015) reported that N proteins induce Treg proliferation. Further investigations have revealed three amino acid regions within the N protein, specifically 15–21 aa, 42–48 aa, and 88–94 aa, which play important roles in inducing Treg proliferation. Reverse genetics approaches have shown that the N^15^ and R^46^ residues in the N protein are crucial for inducing Treg proliferation. These findings contribute to our understanding of the involvement of PRRSV in host immune mechanisms.

PRRSV upregulates cytokine signaling 1 (SOCS1) to modulate the JAK/STAT signaling pathway (Wysocki et al., 2052). The PRRSV N protein can enhance the activity of the suppressor of SOCS1 through its NLS-2 (Luo et al., 2020). The dendritic cell (DC) marker, CD83, is associated with immune suppression, including DC activation and T-cell differentiation. Chen X. et al. (2017) showed that the infection caused by PRRSV triggered the upregulation of soluble CD83 (sCD83) in MoDCs derived from pigs by activating the NF-κB and Sp1 signaling cascades. The N, Nsp10, and Nsp1 proteins can enhance the promoter activity of CD83. Activation of the CD83 promoter relies heavily on two specific amino acids, namely R^43^ and K^44^, within the N protein. The implications of this discovery establish a solid basis for expanding our understanding of the immunosuppressive effects of PRRSV.

NF-κB is important for host cell proliferation and innate immune response. The NF-κB pathway is activated during PRRSV infections. The mechanism underlying this phenomenon was investigated by Luo et al. (2011). They employed NF-κB DNA binding and luciferase activity assays to screen PRRSV structural proteins. NF-κB could be activated by PRRSV N protein. Moreover, the activation of NF-κB by the N protein depended on the sequence spanning from 30–73 aa. DExD/H-box protein 36 (DHX36) is an ATP-dependent RNA helicase. The N protein can enhance the activation of NF-κB through its interaction with the N-terminal tetramer of DHX36 (Jing et al., 2017). These findings provide a basis for understanding PRRSV infections and associated inflammatory responses.

## 9 Application of the N protein in clinical detection

PRRSV antigenic epitopes exist in many non-structural and structural proteins, including the N protein (Ostrowski et al., 2002; Rascón-Castelo et al., 2015). Antibodies against the N protein are easily detected during the initial stages of infection but these antibodies do not neutralize the virus and involved in antibody-dependent enhancement (Murtaugh et al., 2002; Mateu and Diaz, 2007). High levels of this antibody are maintained for several months. PRRSV detection methods rely primarily on assays that target N proteins (Plagemann et al., 2005).

Enzyme-linked immunosorbent Assay (ELISA) is the preferred method for assessing immune responses following vaccination and evaluating the dynamics of PRRSV antibodies over time. This confirms early PRRSV infection and enables the differentiation between antibodies induced by wild-type strains and vaccines (Pan et al., 2023). Cai et al. (2009) developed a PRRSV antigen-capture ELISA method using monoclonal antibodies (mAbs) that have been extensively characterized against North American and European PRRSV N proteins. This antigen detection method can detect purified N proteins from two genotypes, with lower detection limits of 0.4 and 0.8 ng, respectively. Antigen detection methods are valuable field tools for the epidemiological control of PRRSV, allowing for rapid screening, particularly in asymptomatic animals. Zhang et al. (2019) generated three mAbs and identified three epitopes that were recognized by them. The 1G4 monoclonal antibody developed in this study is the first mAb targeting PRRSV-2 N, and amino acid residues 1–15 represent a newly identified epitope of the PRRSV-2 N protein. This discovery may open new perspectives for developing and expanding ELISA detection. Ye et al. (2022) used computational predictions to identify the major antigenic epitopes in the PRRSV N protein. They chemically synthesized three peptides targeting N protein antigenic epitopes as coating antigens and screened the optimal peptide to establish an indirect ELISA method for PRRSV antibody detection. The findings are important for routine diagnosis and epidemiological investigation of PRRSV. SDOW17 is an antibody that recognizes conformational epitopes in the N protein. It is used to detect PRRSV infection in cells. Serine, positioned at residue 90 within the N protein, is pivotal in recognizing conformational epitopes during interactions with SDOW17 mAb (Rappe et al., 2016).

ELISA is continuously being improved and enhanced as a key technology for the clinical detection of PRRSV. Chen et al. (2013) developed an effective indirect ELISA (iELISA) for detecting PRRSV antibodies using the recombinant fusion protein N-Gp5c produced in *Escherichia coli*. This method has been validated by comparison with immunofluorescence analysis and commercial iELISA methods, demonstrating its simplicity and ease of preparation and operation. Duan et al. (2021) developed a competitive ELISA using a nanobody-HRP fusion protein targeting the PRRSV-N protein specifically to detect anti-PRRSV-2 antibodies in pig serum. This method offers several advantages, such as a stable expression system and ease of operation. Sun et al. (2023) selected two high-affinity nanobodies to develop sandwich ELISA. The modified technique showed increased sensitivity. This detection method demonstrated high specificity and could detect all prevalent PRRSV-2 lineages in China.

In addition to ELISA, various detection methods based on the N protein have been established. Song et al. (2006), Chen et al. (2021), and Zhang L. et al. (2022) designed primers and probes for ORF7 of PRRSV to develop RT-qPCR-based detection methods. Hu (2021) developed an immunofluorescence assay (IFA) using the 5B4 monoclonal cell culture supernatant targeting the PRRSV N protein. However, a drawback of IFA is that it relies on viral replication. IFA is prone to nonspecific staining due to multiple interfering factors in the reaction. Luong et al. (2020) developed and conducted a comparative evaluation of the luciferase immunoprecipitation systems (LIPS) assay. The results obtained from LIPS were highly consistent with those of the commercial ELISA. This assay was used to measure the immune reactivity of the serum samples against the N protein and GP3. Compared to GP3, LIPS demonstrated higher diagnostic sensitivity for the N protein. Li et al. (2022) developed an immunochromatographic strip (ICS) based on latex microspheres. As the detection reagent, ICS uses latex microspheres labeled with a specific mAb 1H4 targeting the PRRSV N protein. These results demonstrate that the ICS assay accurately detects PRRSV and shows potential for clinical diagnostic applications.

## 10 Application of N protein-based vaccine

Current commercially available PRRS vaccines have less-than-ideal effectiveness (Nan et al., 2017). A major drawback of these vaccines is their lack of cross-protection. Developing a chimeric vaccine with multiple neutralizing epitopes is a promising strategy for addressing this challenge. Early studies revealed that chimeric viruses constructed by replacing ORF3-6 of VR2332 with the corresponding genes from JA142 or by introducing mutations in ORF2-7 of the FL12 strain to match the LMY sequence can induce cross-reactive neutralizing antibodies (Kim and Yoon, 2008). Targeting ORF7 for vaccine development is a promising strategy.

The N protein undergoes minimal changes during the *in vitro* passage (Liu X. et al., 2014; Chen et al., 2016). The CH-1R vaccine was prepared by inserting the porcine *IL-4* gene between the N and 3′-UTR sequences. This vaccine induces higher levels of IL-4 and a higher proportion of Tregs (Li et al., 2015b). The CH-1R vaccine can incorporate the porcine *GM-CSF* gene at the same position. The recombinant virus induces a higher proportion of Treg cells, elevated levels of IFN-γ, and lower viral viremia (Kimman et al., 2009; Li et al., 2015a). These findings have contributed to the development of novel recombinant vaccines. N proteins possess abundant antigenic epitopes and induce the production of long-lasting non-neutralizing antibodies after PRRSV infection (Plagemann, 2005; Ni et al., 2011), indicating their excellent immunogenicity and reactivity (Ren et al., 2010; Yu et al., 2010). Cheng et al. (2023) produced a mAb, N06, to target the N protein of PRRSV specifically. Using synthetic peptide fragments, they identified a sequential 16 aa pattern that characterized a remarkably immunogenic area within the N protein. mAbs exhibit strong immunogenicity. The immunomodulatory effects of the linearized and truncated forms of the PRRSV-N protein DNA vaccine (pORF7t) primarily manifest in preferentially inducing cellular immune responses against the PRRSV N protein. pORF7t modulates immune responses against PRRSV. Pigs immunized with this vaccine exhibit an increased number of specifically activated Tregs in response to PRRSV infection. This study reveals a novel concept for regulating PRRSV-specific immune responses by inducing cellular immune responses against the N protein, which contributes to developing PRRSV vaccines (Suradhat et al., 2015).

Predicting B and T cell epitopes is important for vaccine design and developing detection methods (El-Manzalawy et al., 2017). The N protein contains four or five antigenic regions (Dea et al., 2000). Although these antigenic regions have been studied extensively, most antigenic epitopes have not been precisely identified (Meulenberg et al., 1998; Plagemann, 2004). Inducing effective and protective B- and T-cell responses, specifically targeting the N protein, is essential for developing innovative vaccine formulations (Luo et al., 2023). Therefore, further experiments focusing on the antigenic epitopes of the N protein should be conducted. Primary and booster immunizations of animals with DNA vaccines encoding truncated N proteins or antigens containing B- and T-cell epitopes from both PRRSV-1 and MLV can induce higher levels of T-cell and antibody responses (Sirisereewan et al., 2016; Bernelin-Cottet et al., 2019). This approach is considered a promising vaccination strategy to enhance the control of PRRS. Song et al. (2011) explored the application of replicon particles in vaccine development. They synthesized a self-replicating, non-propagating replicon RNA *in vitro* and transfected it into cells expressing the N protein. As a result, an infectious particle-packaging replicon RNA was obtained. These generated particles could target a single-round infection and lack intercellular spread. The transposon system targeting PRRSV provides a theoretical basis for studying antiviral vaccines based on viral replicons to prevent PRRSV infection and transmission.

Nanobodies have benefits, including affordability and ease of manufacturing. Duan et al. (2024) investigated the effects of a PRRSV-N-Nb1 nanobody in both laboratory settings and living organisms. Their findings demonstrated that the S105 residue within the PRRSV-N protein functioned as a crucial amino acid that could bind to the R97 residue of PRRSV-N-Nb1. PRRSV-N-Nb1 disrupted the self-interaction of the N protein following viral assembly. This discovery unveils the molecular foundation of N protein self-binding, serving as an essential element in viral replication, and highlights a promising target for advancing antiviral medications against PRRSV.

## 11 Summary and outlook

N protein is a basic phosphorylated protein, and phosphorylation sites are crucial for its biological functions. The N protein possesses an NLS that allows its distribution between the cytoplasm and nucleolus. The N protein can form homodimers participating in RNA genome binding and viral particle assembly. As a structural protein, the PRRSV N protein exhibits high conservation, which has been confirmed by genetic and evolutionary analyses. The PRRSV N protein demonstrates a high nucleotide and amino acid homology level and has been relatively conserved during evolution, with key amino acids being less prone to mutating. Furthermore, disulfide bond formation and the NLS of N proteins are crucial for viral replication. N protein affects PRRSV replication by interacting with PARP-1, DHX9, miR-10a-5p, S100A9, SP1, MOV10, and other factors. Currently, no direct evidence suggests that the N protein affects PRRSV virulence, and further investigation is needed to understand the effects of the N protein on virulence.

The N protein interacts with the PRRSV and its host proteins, enabling diverse biological functions. It interacts with its own GP5, Nsp2, and E proteins and host proteins (including TRIM22, TRIM25, and TRIM26), affecting viral invasion, replication, and signaling pathway regulation. The N protein contains multiple antigenic epitopes, including B- and T-cell epitopes, that form the basis of its involvement in the host immune system. PRRSV N protein regulates the host immune system by inhibiting IFN-I induction, mediating IL-10 production, and activating the NF-κB signaling pathway. Non-neutralizing antibodies induced by the N protein exhibit an early appearance and high levels. The existing PRRSV detection methods are primarily based on N proteins. ELISA methods established using the N protein have been widely used in clinical diagnostics and are continuously optimized and improved. In addition to ELISA, various detection methods based on the N protein have been developed, including RT-qPCR, IFA, LIPS, and ICS. The PRRSV N protein possesses abundant antigenic determinants and exhibits high immunogenicity and conservation, making it a suitable vaccine target. Approaches such as truncating the N protein, inserting exogenous genes, and producing mAbs with good immunogenicity can be used for vaccine development. Nanobody and transposon technologies are novel strategies for developing PRRSV vaccines.

The N protein, a structural protein of PRRSV, is crucial for viral RNA synthesis, viral entry, and the host immune system. Its clinical applications have been extensively studied, and various vaccines and detection technologies targeting the N protein have been developed. The N protein is an important component that should not be overlooked for preventing and treating PRRS. Their extensive biological functions merit further investigation and application in clinical practice.

## Author contributions

YZ: Writing – original draft. GL: Writing – original draft. QL: Writing – original draft. HS: Writing – original draft. HZ: Writing – original draft. RW: Writing – review & editing. WK: Writing – review & editing. JL: Writing – review & editing, Funding acquisition. MZ: Funding acquisition, Writing – review & editing.

## References

[B1] Albataineh M. T. Kadosh D. (2016). Regulatory roles of phosphorylation in model and pathogenic fungi. Med. Mycol. 54, 333–352. 10.1093/mmy/myv098 26705834 PMC4818690

[B2] An T. Q. Li J. N. Su C. M. Yoo D. (2020). Molecular and cellular mechanisms for PRRSV pathogenesis and host response to infection. Virus Res. 286, 197980. 10.1016/j.virusres.2020.197980 32311386 PMC7165118

[B3] An T. Q. Zhou Y. J. Qiu H. J. Tong G. Z. Wang Y. F. Liu J. X.. (2005). Identification of a novel B cell epitope on the nucleocapsid protein of porcine reproductive and respiratory syndrome virus by phage display. Virus Genes 31, 81–87. 10.1007/s11262-005-2203-1 15965612

[B4] Balka G. Wang X. Olasz F. Bálint Á. Kiss I. Bányai K.. (2015). Full genome sequence analysis of a wild, non-MLV-related type 2 Hungarian PRRSV variant isolated in Europe. Virus Res. 200, 1–8. 10.1016/j.virusres.2015.01.014 25616050

[B5] Bao H. Li X. (2021). Emergence and spread of NADC34-like PRRSV in China. Transbound. Emerg. Dis. 68, 3005–3008. 10.1111/tbed.14316 34492162

[B6] Benfield D. A. Nelson E. Collins J. E. Harris L. Goyal S. M. Robison D.. (1992). Characterization of swine infertility and respiratory syndrome (SIRS) virus (isolate ATCC VR-2332). J. Vet. Diagn. Invest. 4, 127–133. 10.1177/104063879200400202 1616976

[B7] Bernelin-Cottet C. Urien C. Stubsrud E. Jakob V. Bouguyon E. Bordet E.. (2019). A DNA-modified live vaccine prime-boost strategy broadens the T-cell response and enhances the antibody response against the porcine reproductive and respiratory syndrome virus. Viruses 11:551. 10.3390/v11060551 31207934 PMC6630347

[B8] Brinton M. Gulyaeva A. Balasuriya U. Dunowska M. Faaberg K. Goldberg T.. (2018). Expansion of the Rank Structure of the Family Arteriviridae and Renaming its Taxa. Available online at: https://talk.ictvonline.org

[B9] Cai J. P. Wang Y. D. Tse H. Xiang H. Yuen K. Y. Che Y. X.. (2009). Detection of asymptomatic antigenemia in pigs infected by porcine reproductive and respiratory syndrome virus (PRRSV) by a novel capture immunoassay with monoclonal antibodies against the nucleocapsid protein of PRRSV. Clin. Vaccine Immunol. 16, 1822–1828. 10.1128/CVI.00244-09 19828768 PMC2786387

[B10] Cao Y. Ouyang H. Zhang M. Chen F. Yang X. Pang D.. (2014). Analysis of molecular variation in porcine reproductive and respiratory syndrome virus in China between 2007 and 2012. Virol. Sin. 29, 183–188. 10.1007/s12250-014-3462-6 24950785 PMC8206242

[B11] Chand R. J. Trible B. R. Rowland R. R. (2012). Pathogenesis of porcine reproductive and respiratory syndrome virus. Curr. Opin. Virol. 2, 256–263. 10.1016/j.coviro.2012.02.002 22709514

[B12] Chen C. Fan W. Jia X. Li J. Bi Y. Liu W.. (2013). Development of a recombinant N-Gp5c fusion protein-based ELISA for detection of antibodies to porcine reproductive and respiratory syndrome virus. J Virol Methods. 189, 213–220. 10.1016/j.jviromet.2013.02.003 23439032

[B13] Chen J. Shi X. Zhang X. Wang A. Wang L. Yang Y.. (2017). MicroRNA 373 facilitates the replication of porcine reproductive and respiratory syndrome virus by its negative regulation of type I interferon induction. J. Virol. 91:3. 10.1128/JVI.01311-16 27881653 PMC5244336

[B14] Chen J. Zhao S. Cui Z. Li W. Xu P. Liu H.. (2022). MicroRNA-376b-3p promotes porcine reproductive and respiratory syndrome virus replication by targeting viral restriction factor TRIM22. J. Virol. 96, e0159721. 10.1128/JVI.01597-21 34757838 PMC8791308

[B15] Chen L. Keppler O. T. Schölz C. (2018). Post-translational Modification-Based Regulation of HIV Replication. Front. Microbiol. 9, 2131. 10.3389/fmicb.2018.02131 30254620 PMC6141784

[B16] Chen X. Zhang Q. Bai J. Zhao Y. Wang X. Wang H.. (2017). The nucleocapsid protein and nonstructural protein 10 of highly pathogenic porcine reproductive and respiratory syndrome virus enhance CD83 production via NF-κB and Sp1 signaling pathways. J. Virol. 91:18. 10.1128/JVI.00986-17 28659471 PMC5571251

[B17] Chen Y. He S. Sun L. Luo Y. Sun Y. Xie J.. (2016). Genetic variation, pathogenicity, and immunogenicity of highly pathogenic porcine reproductive and respiratory syndrome virus strain XH-GD at different passage levels. Arch. Virol. 161, 77–86. 10.1007/s00705-015-2597-6 26483282

[B18] Chen Y. Shi K. Liu H. Yin Y. Zhao J. Long F.. (2021). Development of a multiplex qRT-PCR assay for detection of African swine fever virus, classical swine fever virus and porcine reproductive and respiratory syndrome virus. J. Vet. Sci. 22, e87. 10.4142/jvs.2021.22.e87 34854269 PMC8636662

[B19] Chen Y. Xing X. Li Q. Feng S. Han X. He S.. (2018). Serine 105 and 120 are important phosphorylation sites for porcine reproductive and respiratory syndrome virus N protein function. Vet. Microbiol. 219, 128–135. 10.1016/j.vetmic.2018.04.010 29778185 PMC7117435

[B20] Chen Y. Yu Z. Yi H. Wei Y. Han X. Li Q.. (2019). The phosphorylation of the N protein could affect PRRSV virulence *in vivo*. Vet. Microbiol. 231, 226–231. 10.1016/j.vetmic.2019.03.018 30955814 PMC7117339

[B21] Cheng Y. Wu M. Xiao L. Zhang M. Huang B. Cong F.. (2023). Identificationof a novel linear epitope on the porcine reproductive and respiratory syndrome virus nucleocapsid protein, as recognized by a specific monoclonal antibody. Front. Immunol. 14:1165396. 10.3389/fimmu.2023.1165396 37143683 PMC10151797

[B22] Collins J. E. Benfield D. A. Christianson W. T. Harris L. Hennings J. C. Shaw D. P.. (1992). Isolation of swine infertility and respiratory syndrome virus (isolate ATCC VR-2332) in North America and experimental reproduction of the disease in gnotobiotic pigs. J. Vet. Diagn. Invest. 4, 117–126. 10.1177/104063879200400201 1616975

[B23] Dea S. Gagnon C. A. Mardassi H. Pirzadeh B. Rogan D. (2000). Current knowledge on the structural proteins of porcine reproductive and respiratory syndrome (PRRS) virus: comparison of the North American and European isolates. Arch. Virol. 145, 659–688. 10.1007/s007050050662 10893147 PMC7087215

[B24] Doan D. N. Dokland T. (2003). Structure of the nucleocapsid protein of porcine reproductive and respiratory syndrome virus. Structure 11, 1445–1451. 10.1016/j.str.2003.09.018 14604534 PMC7172083

[B25] Dokland T. (2010). The structural biology of PRRSV. Virus Res. 154, 86–97. 10.1016/j.virusres.2010.07.029 20692304 PMC7114433

[B26] Dokland T. Walsh M. Mackenzie J. M. Khromykh A. A. Ee K. H. Wang S.. (2004). West Nile virus core protein; tetramer structure and ribbon formation. Structure 12, 1157–1163. 10.1016/j.str.2004.04.024 15242592 PMC7173237

[B27] Du T. Nan Y. Xiao S. Zhao Q. Zhou M. E. (2017). Antiviral Strategies against PRRSV Infection. Trends Microbiol. 25, 968–979. 10.1016/j.tim.2017.06.001 28652073

[B28] Duan H. Chen X. Zhang Z. Zhang Z. Li Z. Wang X.. (2024). A nanobody inhibiting porcine reproductive and respiratory syndrome virus replication via blocking self-interaction of viral nucleocapsid protein. J. Virol. 23:e0131923. 10.1128/jvi.01319-23 38084961 PMC10804987

[B29] Duan H. Chen X. Zhao J. Zhu J. Zhang G. Fan M.. (2021). Development of a nanobody-based competitive enzyme-linked immunosorbent assay for efficiently and specifically detecting antibodies against genotype 2 porcine reproductive and respiratory syndrome viruses. J. Clin. Microbiol. 59, e0158021. 10.1128/JCM.01580-21 34524888 PMC8601240

[B30] El-Manzalawy Y. Dobbs D. Honavar G. V. (2017). In silico prediction of linear B-cell epitopes on proteins. Methods Mol. Biol. 1484, 255–264. 10.1007/978-1-4939-6406-2_17 27787831 PMC8109234

[B31] Fahad M. I. Kapil S. (2001). Interactions of cellular proteins with the positive strand of 3′-untranslated region RNA and the nucleoprotein gene of porcine reproductive and respiratory syndrome virus. Adv. Exp. Med. Biol. 494, 633–639. 10.1007/978-1-4615-1325-4_94 11774538

[B32] Fan B. Liu X. Bai J. Li Y. Zhang Q. Jiang P.. (2015). The 15N and 46R residues of highly pathogenic porcine reproductive and respiratory syndrome virus nucleocapsid protein enhance regulatory T lymphocytes proliferation. PLoS ONE 10, e0138772. 10.1371/journal.pone.0138772 26397116 PMC4580451

[B33] Fang J. Wang H. Bai J. Zhang Q. Li Y. Liu F.. (2016). Monkey viperin restricts porcine reproductive and respiratory syndrome virus replication. PLoS ONE 11, e0156513. 10.1371/journal.pone.0156513 27232627 PMC4883763

[B34] Fang Y. Snijder J. E. (2010). The PRRSV replicase: exploring the multifunctionality of an intriguing set of nonstructural proteins. Virus Res. 154:61–76. 10.1016/j.virusres.2010.07.030 20696193 PMC7114499

[B35] Fang Y. Treffers E. E. Li Y. Tas A. Sun Z. van der Meer Y.. (2012). Efficient−2 frameshifting by mammalian ribosomes to synthesize an additional arterivirus protein. Proc. Natl. Acad. Sci. USA. 109, E2920–E2928. 10.1073/pnas.1211145109 23043113 PMC3491471

[B36] Forsberg R. (2005). Divergence time of porcine reproductive and respiratory syndrome virus subtypes. Mol. Biol. Evol. 22, 2131–2134. 10.1093/molbev/msi208 16000650

[B37] Fu Y. Quan R. Zhang H. Hou J. Tang J. Feng H. W.. (2012). Porcine reproductive and respiratory syndrome virus induces interleukin-15 through the NF-κB signaling pathway. J. Virol. 86, 7625–7636. 10.1128/JVI.00177-12 22573868 PMC3416278

[B38] Gall L. Legeay O. Bourhy H. Arnauld C. Albina E. Jestin A. (1998). Molecular variation in the nucleoprotein gene (ORF7) of the porcine reproductive and respiratory syndrome virus (PRRSV). Virus Res. 54, 9–21. 10.1016/S0168-1702 9660067

[B39] Goldman R. D. Khuon S. Chou Y. H. Opal P. Steinert M. P. (1996). The function of intermediate filaments in cell shape and cytoskeletal integrity. J. Cell Biol. 134, 971–983. 10.1083/jcb.134.4.971 8769421 PMC2120965

[B40] Guo Z. Chen X. X. Li R. Qiao S. Zhang G. (2018). The prevalent status and genetic diversity of porcine reproductive and respiratory syndrome virus in China: a molecular epidemiological perspective. Virol. J. 15:2. 10.1186/s12985-017-0910-6 29301547 PMC5753475

[B41] Han M. Yoo D. (2014). Modulation of innate immune signaling by nonstructural protein 1 (nsp1) in the family Arteriviridae. Virus Res. 194, 100–109. 10.1016/j.virusres.2014.09.007 25262851 PMC7114407

[B42] Holtkamp D. Kliebenstein J. Neumann E. Zimmerman J. Rotto H. Yoder Y.. (2005). Assessment of the economic impact of porcine reproductive 581 and respiratory syndrome virus on United States pork producers. J. Am. Vet. Med. Assoc. 21, 72–84 10.2460/javma.2005.227.385 16121604

[B43] Hou J. Wang L. Quan R. Fu Y. Zhang H. Feng H. W.. (2012). Induction of interleukin-10 is dependent on p38 mitogen-activated protein kinase pathway in macrophages infected with porcine reproductive and respiratory syndrome virus. Virol. J. 9, 165. 10.1186/1743-422X-9-165 22909062 PMC3441385

[B44] Hu Y. (2021). Generation of Monoclonal Antibody Against PRRSV HeB,08 Strain and Establishment of TFA Method, Master.

[B45] Jing H. Song Y. Li H. Duan E. Liu J. Ke W.. (2023). HnRNP K reduces viral gene expression by targeting cytosine-rich sequences in porcine reproductive and respiratory syndrome virus-2 genome to dampen the viral growth. Virology 581, 15–25. 10.1016/j.virol.2023.02.006 36842269

[B46] Jing H. Tao R. Dong N. Cao S. Sun Y. Ke W.. (2019). Nuclear localization signal in TRIM22 is essential for inhibition of type 2 porcine reproductive and respiratory syndrome virus replication in MARC-145 cells. Virus Genes 55, 660–672. 10.1007/s11262-019-01691-x 31375995 PMC7089487

[B47] Jing H. Zhou Y. Fang L. Ding Z. Wang D. Ke W.. (2017). DExD/H-box helicase 36 signaling via myeloid differentiation primary response gene 88 contributes to NF-κB activation to type 2 porcine reproductive and respiratory syndrome virus infection. Front. Immunol. 8, 1365. 10.3389/fimmu.2017.01365 29123520 PMC5662876

[B48] Jourdan S. S. Osorio F. A. Hiscox A. J. (2011). Biophysical characterisation of the nucleocapsid protein from a highly pathogenic porcine reproductive and respiratory syndrome virus strain. Biochem. Biophys. Res. Commun. 419, 137–141. 10.1016/j.bbrc.2011.11.126 22306009 PMC7092862

[B49] Ke H. Lee S. Kim J. Liu H. C. Yoo D. (2019). Interaction of PIAS1 with PRRS virus nucleocapsid protein mediates NF-κB activation and triggers proinflammatory mediators during viral infection. Sci. Rep. 9, 11042. 10.1038/s41598-019-47495-9 31363150 PMC6667501

[B50] Ke H. Yoo D. (2017). The viral innate immune antagonism and an alternative vaccine design for PRRS virus. Vet. Microbiol. 209, 75–89. 10.1016/j.vetmic.2017.03.014 28341332 PMC7111430

[B51] Kenney S. P. Meng J. X. (2015). An SH3 binding motif within the nucleocapsid protein of porcine reproductive and respiratory syndrome virus interacts with the host cellular signaling proteins STAMI, TXK, Fyn, Hck, and cortactin. Virus Res. 204, 31–39. 10.1016/j.virusres.2015.04.004 25882913

[B52] Kim W. I. Yoon J. K. (2008). Molecular assessment of the role of envelope-associated structural proteins in cross neutralization among different PRRS viruses. Virus Genes 37, 380–391. 10.1007/s11262-008-0278-1 18770017

[B53] Kimman T. G. Cornelissen L. A. Moormann R. J. Rebel J. M. Stockhofe-Zurwieden N. (2009). Challenges for porcine reproductive and respiratory syndrome virus (PRRSV) vaccinology. Vaccine, 27(Wootton et al., 2002) 3704-18 (2009) 10.1016/j.vaccine.2009.04.022 19464553

[B54] Krol J. Loedige I. Filipowicz W. (2010). The widespread regulation of microRNA biogenesis, function and decay. Nat. Rev. Genet. 11, 597–610. 10.1038/nrg2843 20661255

[B55] Kwon B. Ansari I. H. Pattnaik A. K. Osorio A. F. (2008). Identification of virulence determinants of porcine reproductive and respiratory syndrome virus through construction of chimeric clones. Virology 380, 371–378. 10.1016/j.virol.2008.07.030 18768197

[B56] Lalonde C. Provost C. Gagnon A. C. (2020). Whole-genome sequencing of porcine reproductive and respiratory syndrome virus from field clinical samples improves the genomic surveillance of the virus. J. Clin. Microbiol. 58:20. 10.1128/JCM.00097-20 32817228 PMC7587098

[B57] Lee C. Calvert J. G. Welch S. K. Yoo D. (2004). A DNA-launched reverse genetics system for porcine reproductive and respiratory syndrome virus reveals that homodimerization of the nucleocapsid protein is essential for virus infectivity. Virology 331, 47–62. 10.1016/j.virol.2004.10.026 15582652

[B58] Lee C. Hodgins D. Calvert J. G. Welch S. K. Jolie R. Yoo D.. (2005). Mutations within the nuclear localization signal of the porcine reproductive and respiratory syndrome virus nucleocapsid protein attenuate virus replication. Virology 346, 238–250. 10.1016/j.virol.2005.11.005 16330065 PMC7172752

[B59] Lee C. Yoo D. (2005). Cysteine residues of the porcine reproductive and respiratory syndrome virus small envelope protein are non-essential for virus infectivity. J. Gen. Virol. 86, 3091–3096. 10.1099/vir.0.81160-0 16227232

[B60] Lee S. M. Kleiboeker B. S. (2005). Porcine arterivirus activates the NF-kappaB pathway through IkappaB degradation. Virology 342, 47–59. 10.1016/j.virol.2005.07.034 16129468 PMC7111765

[B61] Li C. Zhuang J. Wang J. Han L. Sun Z. Xiao Y.. (2016). Outbreak Investigation of NADC30-Like PRRSV in South-East China. Transbound. Emerg. Dis. 63, 474–479. 10.1111/tbed.12530 27292168

[B62] Li W. Li M. Zhang H. Li C. Xu H. Gong B.. (2022). A novel immunochromatographic strip based on latex microspheres for the rapid detection of north american-type porcine reproductive and respiratory syndrome virus. Front. Microbiol. 13:882112. 10.3389/fmicb.2022.882112 35572691 PMC9100670

[B63] Li Y. Firth A. E. Brierley I. Cai Y. Napthine S. Wang T.. (2019). Programmed−2/-1 ribosomal frameshifting in simarteriviruses: an evolutionarily conserved mechanism. J. Virol. 93:16. 10.1128/JVI.00370-19 31167906 PMC6675879

[B64] Li Z. Wang G. Wang Y. Zhang C. Huang B. Li Q.. (2015a). Immune responses of pigs immunized with a recombinant porcine reproductive and respiratory syndrome virus expressing porcine GM-CSF. Vet. Immunol. Immunopathol. 168, 40–48. 10.1016/j.vetimm.2015.08.003 26300317

[B65] Li Z. Wang G. Wang Y. Zhang C. Wang X. Huang B.. (2015b). Rescue and evaluation of a recombinant PRRSV expressing porcine Interleukin-4. Virol. J. 12, 185. 10.1186/s12985-015-0380-7 26573719 PMC4647277

[B66] Liu L. Lear Z. Hughes D. J. Wu W. Zhou E. M. Whitehouse A.. (2014). Resolution of the cellular proteome of the nucleocapsid protein from a highly pathogenic isolate of porcine reproductive and respiratory syndrome virus identifies PARP-1 as a cellular target whose interaction is critical for virus biology. Vet. Microbiol. 176, 109–119. 10.1016/j.vetmic.2014.11.023 25614100 PMC4414928

[B67] Liu L. Tian J. Nan H. Tian M. Li Y. Xu X.. (2016). Porcine reproductive and respiratory syndrome virus nucleocapsid protein interacts with Nsp9 and cellular DHX9 to regulate viral RNA synthesis. J. Virol. 90, 5384–5398. 10.1128/JVI.03216-15 27009951 PMC4934760

[B68] Liu X. Fan B. Bai J. Wang H. Li Y. Jiang J.. (2015). The N-N non-covalent domain of the nucleocapsid protein of type 2 porcine reproductive and respiratory syndrome virus enhances induction of IL-10 expression. J. Gen. Virol. 96, 1276–1286. 10.1099/vir.0.000061 25614594

[B69] Liu X. Li Y. Lu Q. Bai J. Wang X. Jiang P.. (2014). A new porcine reproductive and respiratory syndrome virus strain with highly conserved molecular characteristics in its parental and attenuated strains. Virus Genes 49, 259–268. 10.1007/s11262-014-1086-4 24859421

[B70] Lunney J. K. Fang Y. Ladinig A. Chen N. Li Y. Rowland B.. (2016). Porcine reproductive and respiratory syndrome virus (PRRSV): pathogenesis and Interaction with the immune system. Annu Rev Anim Biosci 4, 129–154. 10.1146/annurev-animal-022114-111025 26646630

[B71] Luo Q. Zheng Y. Zhang H. Yang Z. Sha H. Kong W.. (2023). Research progress on glycoprotein 5 of porcine reproductive and respiratory syndrome virus. Animals (Basel) 13:5. 10.3390/ani13050813 36899670 PMC10000246

[B72] Luo R. Fang L. Jiang Y. Jin H. Wang Y. Wang D.. (2011). Activation of NF-κB by nucleocapsid protein of the porcine reproductive and respiratory syndrome virus. Virus Genes 42, 76–81. 10.1007/s11262-010-0548-6 21063763

[B73] Luo X. Chen X. X. Qiao S. Li R. Xie S. Zhou X.. (2020). Porcine reproductive and respiratory syndrome virus enhances self-replication via AP-1-dependent induction of SOCS1. J. Immunol. 204, 394–407. 10.4049/jimmunol.1900731 31826939 PMC6943376

[B74] Luong H. Q. Lai H. T. L. Vu H. (2020). Evaluation of antibody response directed against porcine reproductive and respiratory syndrome virus structural proteins. Vaccines (Basel) 8:3. 10.3390/vaccines8030533 32947931 PMC7564207

[B75] Mardassi H. Athanassious R. Mounir S. Dea S. (1994). Porcine reproductive and respiratory syndrome virus: morphological, biochemical and serological characteristics of Quebec isolates associated with acute and chronic outbreaks of porcine reproductive and respiratory syndrome. Can. J. Vet. Res. 58, 55–64. 8143254 PMC1263660

[B76] Mardassi H. Mounir S. Dea S. (1995). Molecular analysis of the ORFs 3 to 7 of porcine reproductive and respiratory syndrome virus, Québec reference strain. Arch. Virol. 140, 1405–1418. 10.1007/BF01322667 7661693 PMC7087035

[B77] Mateu E. Diaz I. (2007). The challenge of PRRS immunology. Vet J. 177, 345–351. 10.1016/j.tvjl.2007.05.022 17644436 PMC7110845

[B78] Meng X. J. (2000). Heterogeneity of porcine reproductive and respiratory syndrome virus: implications for current vaccine efficacy and future vaccine development. Vet. Microbiol. 74, 309–329. 10.1016/S0378-1135(00)00196-6 10831854 PMC7117501

[B79] Meng X. J. Paul P. S. Halbur P. G. Lum A. M. (1995). Phylogenetic analyses of the putative M (ORF 6) and N (ORF 7) genes of porcine reproductive and respiratory syndrome virus (PRRSV): implication for the existence of two genotypes of PRRSV in the U.S.A. and Europe. Arch. Virol. 140, 745–755. 10.1007/BF01309962 7794115 PMC7086766

[B80] Meulenberg J. J. Petersen-den Besten A. de Kluyver E. P. Moormann R. J. Schaaper W. M. Wensvoort G. (1995). Characterization of structural proteins of Lelystad virus. Adv. Exp. Med. Biol. 380, 271–276. 10.1007/978-1-4615-1899-0_43 8830491

[B81] Meulenberg J. J. van Nieuwstadt A. P. van Essen-Zandbergen A. (1998). Bos-de Ruijter JN, Langeveld JP, Meloen HR: Localization and fine mapping of antigenic sites on the nucleocapsid protein N of porcine reproductive and respiratory syndrome virus with monoclonal antibodies. Virology 252, 106–114. 10.1006/viro.1998.9436 9875321

[B82] Montaner-Tarbes S. Del Portillo H. A. Montoya M. Fraile L. (2019). Key gaps in the knowledge of the porcine respiratory reproductive syndrome virus (PRRSV). Front. Vet. Sci. 6, 38. 10.3389/fvets.2019.00038 30842948 PMC6391865

[B83] Murtaugh M. P. Xiao Z. Zuckermann F. (2002). Immunological responses of swine to porcine reproductive and respiratory syndrome virus infection. Viral Immunol. 15, 533–547. 10.1089/088282402320914485 12513925

[B84] Music N. Gagnon A. C. (2010). The role of porcine reproductive and respiratory syndrome (PRRS) virus structural and non-structural proteins in virus pathogenesis. Anim. Health Res. Rev. 11, 135–163. 10.1017/S1466252310000034 20388230

[B85] Nan Y. Wu C. Gu G. Sun W. Zhang Y. J. Zhou M. E.. (2017). Improved vaccine against PRRSV: current progress and future perspective. Front. Microbiol. 8:1635. 10.3389/fmicb.2017.01635 28894443 PMC5581347

[B86] Ni Y. Y. Huang Y. W. Cao D. Opriessnig T. Meng J. X. (2011). Establishment of a DNA-launched infectious clone for a highly pneumovirulent strain of type 2 porcine reproductive and respiratory syndrome virus: identification and in vitro and in vivo characterization of a large spontaneous deletion in the nsp2 region. Virus Res. 160, 264–273. 10.1016/j.virusres.2011.06.027 21763365

[B87] Ostrowski M. Galeota J. A. Jar A. M. Platt K. B. Osorio F. A. Lopez J. O.. (2002). Identification of neutralizing and nonneutralizing epitopes in the porcine reproductive and respiratory syndrome virus GP5 ectodomain. J. Virol. 76, 4241–4250. 10.1128/JVI.76.9.4241-4250.2002 11932389 PMC155073

[B88] Pan J. Zeng M. Zhao M. Huang L. (2023). Research progress on the detection methods of porcine reproductive and respiratory syndrome virus. Front. Microbiol. 14, 1097905. 10.3389/fmicb.2023.1097905 36970703 PMC10033578

[B89] Pei Y. Hodgins D. C. Lee C. Calvert J. G. Welch S. K. Jolie R.. (2008). Functional mapping of the porcine reproductive and respiratory syndrome virus capsid protein nuclear localization signal and its pathogenic association. Virus Res. 135, 107–14. 10.1016/j.virusres.2008.02.012 18403041

[B90] Plagemann P. G. (2004). Epitope specificity of monoclonal antibodies to the N-protein of porcine reproductive and respiratory syndrome virus determined by ELISA with synthetic peptides. Vet Immunol Immunopathol. 104, 59–68. 10.1016/j.vetimm.2004.10.004 15661331

[B91] Plagemann P. G. (2005). Peptide ELISA for measuring antibodies to N-protein of porcine reproductive and respiratory syndrome virus. J Virol Methods 134, 99–118. 10.1016/j.jviromet.2005.12.003 16426684

[B92] Plagemann P. G. Rowland R. R. Cafruny A. W. (2005). Polyclonal hypergammaglobulinemia and formation of hydrophobic immune complexes in porcine reproductive and respiratory syndrome virus-infected and uninfected pigs. Viral Immunol. 18, 138–147. 10.1089/vim.2005.18.138 15802958

[B93] Rappe J. C. García-Nicolás O. Flückiger F. Thür B. Hofmann M. A. Summerfield A.. (2016). Heterogeneous antigenic properties of the porcine reproductive and respiratory syndrome virus nucleocapsid. Vet. Res. 47, 117. 10.1186/s13567-016-0399-9 27871316 PMC5118883

[B94] Rascón-Castelo E. Burgara-Estrella A. Mateu E. Hernández J. (2015). Immunological features of the non-structural proteins of porcine reproductive and respiratory syndrome virus. Viruses 7, 873–886. 10.3390/v7030873 25719944 PMC4379552

[B95] Ren X. Wang M. Yin J. Li G. (2010). Phages harboring specific peptides that recognize the N protein of the porcine reproductive and respiratory syndrome virus distinguish the virus from other viruses. J. Clin. Microbiol. 48, 1875–1881. 10.1128/JCM.01707-09 20237096 PMC2863871

[B96] Renken C. Nathues C. Swam H. Fiebig K. Weiss C. Eddicks M.. (2021). Application of an economic calculator to determine the cost of porcine reproductive and respiratory syndrome at farm-level in 21 pig herds in Germany. Porcine Health Manag. 7:3. 10.1186/s40813-020-00183-x 33397503 PMC7784293

[B97] Rowland R. R. Kervin R. Kuckleburg C. Sperlich A. Benfield A. D. (1999). The localization of porcine reproductive and respiratory syndrome virus nucleocapsid protein to the nucleolus of infected cells and identification of a potential nucleolar localization signal sequence. Virus Res. 64, 1–12. 10.1016/S0168-1702(Sun_et_al.,_2012)00048-9 10500278

[B98] Rowland R. R. Schneider P. Fang Y. Wootton S. Yoo D. Benfield A. D.. (2003). Peptide domains involved in the localization of the porcine reproductive and respiratory syndrome virus nucleocapsid protein to the nucleolus. Virology 316, 135–145. 10.1016/S0042-6822(03)00482-3 14599798 PMC7125632

[B99] Rowland R. R. Yoo D. (2003). Nucleolar-cytoplasmic shuttling of PRRSV nucleocapsid protein: a simple case of molecular mimicry or the complex regulation by nuclear import, nucleolar localization and nuclear export signal sequences. Virus Res. 95, 23–33. 10.1016/S0168-1702(03)00161-8 12921993 PMC7127199

[B100] Sagong M. Lee C. (2010). Differential cellular protein expression in continuous porcine alveolar macrophages regulated by the porcine reproductive and respiratory syndrome virus nucleocapsid protein. Virus Res. 151, 88–96. 10.1016/j.virusres.2010.04.003 20394785

[B101] Sagong M. Lee C. (2011). Porcine reproductive and respiratory syndrome virus nucleocapsid protein modulates interferon-β production by inhibiting IRF3 activation in immortalized porcine alveolar macrophages. Arch. Virol. 156, 2187–2195. 10.1007/s00705-011-1116-7 21947566 PMC7086947

[B102] Shi C. Liu Y. Ding Y. Zhang Y. Zhang J. (2015). PRRSV receptors and their roles in virus infection. Arch. Microbiol. 197, 503–512. 10.1007/s00203-015-1088-1 25666932

[B103] Sirisereewan C. Nedumpun T. Kesdangsakonwut S. Woonwong Y. Kedkovid R. Arunorat J.. (2016). Positive immunomodulatory effects of heterologous DNA vaccine- modified live vaccine, prime-boost immunization, against the highly-pathogenic PRRSV infection. Vet. Immunol. Immunopathol. 183, 7–15. 10.1016/j.vetimm.2016.11.002 28063479

[B104] Snijder E. J. Kikkert M. Fang Y. (2013). Arterivirus molecular biology and pathogenesis. J. Gen Virol. 94, 2141–2163. 10.1099/vir.0.056341-0 23939974

[B105] Song B. H. Kim J. M. Kim J. K. Jang H. S. Yun G. N. Choi E. J.. (2011). Packaging of porcine reproductive and respiratory syndrome virus replicon RNA by a stable cell line expressing its nucleocapsid protein. J. Microbiol. 49, 516–523. 10.1007/s12275-011-1280-1 21717343 PMC7091078

[B106] Song C. Lu R. Bienzle D. Liu H. C. Yoo D. (2009). Interaction of the porcine reproductive and respiratory syndrome virus nucleocapsid protein with the inhibitor of MyoD family-a domain-containing protein. Biol. Chem. 390, 215–223. 10.1515/BC.2009.028 19090724

[B107] Song T. Fang L. Wang D. Zhang R. Zeng S. An K.. (2016). Quantitative interactome reveals that porcine reproductive and respiratory syndrome virus nonstructural protein 2 forms a complex with viral nucleocapsid protein and cellular vimentin. J. Proteomics 142, 70–81. 10.1016/j.jprot.2016.05.009 27180283

[B108] Song Y. Guo Y. Li X. Sun R. Zhu M. Shi J.. (2021). RBM39 Alters phosphorylation of c-Jun and binds to viral RNA to promote PRRSV proliferation. Front. Immunol. 12, 664417. 10.3389/fimmu.2021.664417 34079549 PMC8165236

[B109] Song Z. Bai J. Liu X. Nauwynck H. Wu J. Liu X.. (2019). S100A9 regulates porcine reproductive and respiratory syndrome virus replication by interacting with the viral nucleocapsid protein. Vet. Microbiol. 239, 108498. 10.1016/j.vetmic.2019.108498 31767072 PMC7125916

[B110] Song Z. Song C. Yang Z. Zhu D. Wu Z. Jiang Z.. (2006). Development of real-time TapMan-quantitative RT-PCR assay for detection of porcine reproductive and respiratory syndrome virus. Vet. Sci. Chin(02), 98-102 (2006)

[B111] Sun M. Sun Y. Yang Y. Zhao M. Cao D. Zhang M.. (2023). Multivalent nanobody-based sandwich enzyme-linked immunosorbent assay for sensitive detection of porcine reproductive and respiratory syndrome virus. Int. J. Biol. Macromol. 258, 128896. 10.1016/j.ijbiomac.2023.128896 38143067

[B112] Sun Q. Xu H. Li C. Gong B. Li Z. Tian Z. J.. (2022). Emergence of a novel PRRSV-1 strain in mainland China: A recombinant strain derived from the two commercial modified live viruses Amervac and DV. Front. Vet. Sci. 9, 974743. 10.3389/fvets.2022.974743 36157177 PMC9505512

[B113] Sun Y. Han M. Kim C. Calvert J. G. Yoo D. (2012). Interplay between interferon-mediated innate immunity and porcine reproductive and respiratory syndrome virus. Viruses 4, 424–446. 10.3390/v4040424 22590680 PMC3347317

[B114] Suradhat S. Wongyanin P. Kesdangsakonwut S. Teankum K. Lumyai M. Triyarach S.. (2015). A novel DNA vaccine for reduction of PRRSV-induced negative immunomodulatory effects: A proof of concept. Vaccine 33, 3997–4003. 10.1016/j.vaccine.2015.06.020 26079617

[B115] Veit M. Matczuk A. K. Sinhadri B. C. Krause E. Thaa B. (2014). Membrane proteins of arterivirus particles: structure, topology, processing and function. Virus Res. 194, 16–36. 10.1016/j.virusres.2014.09.010 25278143 PMC7172906

[B116] Vizcaíno C. Mansilla S. Portugal J. (2015). Sp1 transcription factor: a long-standing target in cancer chemotherapy. Pharmacol. Ther. 152, 111–124. 10.1016/j.pharmthera.2015.05.008 25960131

[B117] Wang C. Zeng N. Liu S. Miao Q. Zhou L. Ge X.. (2017). Interaction of porcine reproductive and respiratory syndrome virus proteins with SUMO-conjugating enzyme reveals the SUMOylation of nucleocapsid protein. PLoS ONE 12, e0189191. 10.1371/journal.pone.0189191 29236778 PMC5728522

[B118] Wang H. Bai J. Fan B. Li Y. Zhang Q. Jiang P.. (2016). The interferon-induced Mx2 inhibits porcine reproductive and respiratory syndrome virus replication. J. Interferon Cytokine Res. 36, 129–139. 10.1089/jir.2015.0077 26566027

[B119] Wang L. J. Wan B. Guo Z. Qiao S. Li R. Xie S.. (2018). Genomic analysis of a recombinant NADC30-like porcine reproductive and respiratory syndrome virus in china. Virus Genes 54, 86–97. 10.1007/s11262-017-1516-1 29090410

[B120] Wang L. J. Xie W. Chen X. X. Qiao S. Zhao M. Gu Y.. (2017). Molecular epidemiology of porcine reproductive and respiratory syndrome virus in Central China since 2014: The prevalence of NADC30-like PRRSVs. Microb. Pathog. 109, 20–28. 10.1016/j.micpath.2017.05.021 28512020

[B121] Wang Q. Peng J. Sun Y. Chen J. An T. Leng C.. (2014). Unique epitopes recognized by monoclonal antibodies against HP-PRRSV: deep understanding of antigenic structure and virus-antibody interaction. PLoS ONE 9, e111633. 10.1371/journal.pone.0111633 25360600 PMC4216098

[B122] Wang Q. Yi H. Guo Y. Sun Y. Yu Z. Lu L.. (2023). PCNA promotes PRRSV replication by increasing the synthesis of viral genome. Vet. Microbiol. 281, 109741. 10.1016/j.vetmic.2023.109741 37087878

[B123] Wang R. Nan Y. Yu Y. Yang Z. Zhang J. Y. (2013). Variable interference with interferon signal transduction by different strains of porcine reproductive and respiratory syndrome virus. Vet Microbiol. 166, 493–503. 10.1016/j.vetmic.2013.07.022 23953026

[B124] Wang W. W. Zhang L. Ma X. C. Gao J. M. Xiao Y. H. Zhou M. E.. (2011). The role of vimentin during PRRSV infection of Marc-145 cells. Bing Du Xue Bao 27, 456–461. 10.13242/j.cnki.bingduxuebao.002205 21998958

[B125] Wang X. Bai J. Zhang L. Wang X. Li Y. Jiang P.. (2012). Poly(A)-binding protein interacts with the nucleocapsid protein of porcine reproductive and respiratory syndrome virus and participates in viral replication. Antiviral. Res. 96, 315–323. 10.1016/j.antiviral.2012.09.004 22985629

[B126] Wang Y. Liang Y. Han J. Burkhart K. M. Vaughn E. M. Roof M. B.. (2007). Attenuation of porcine reproductive and respiratory syndrome virus strain MN184 using chimeric construction with vaccine sequence. Virology 371, 418–29. 10.1016/j.virol.2007.09.032 17976680

[B127] Wensvoort G. Terpstra C. Pol J. M. (1991). ter Laak EA, Bloemraad M, de Kluyver EP, et al.: Mystery swine disease in The Netherlands: the isolation of Lelystad virus. Vet. Q. 13, 121–130. 10.1080/01652176.1991.9694296 1835211

[B128] Wongyanin P. Buranapraditkul S. Yoo D. Thanawongnuwech R. Roth J. A. Suradhat S.. (2012). Role of porcine reproductive and respiratory syndrome virus nucleocapsid protein in induction of interleukin-10 and regulatory T-lymphocytes (Treg). J. Gen. Virol. 93, 1236–1246. 10.1099/vir.0.040287-0 22422061

[B129] Wootton S. Koljesar G. Yang L. Yoon K. J. Yoo D. (2001). Antigenic importance of the carboxy-terminal beta-strand of the porcine reproductive and respiratory syndrome virus nucleocapsid protein. Clin. Diagn. Lab. Immunol. 8, 598–603. 10.1128/CDLI.8.3.598-603.2001 11329465 PMC96108

[B130] Wootton S. K. Nelson E. A. Yoo D. (1998). Antigenic structure of the nucleocapsid protein of porcine reproductive and respiratory syndrome virus. Clin. Diagn. Lab. Immunol. 5, 773–779. 10.1128/CDLI.5.6.773-779.1998 9801333 PMC96200

[B131] Wootton S. K. Rowland R. R. Yoo D. (2002). Phosphorylation of the porcine reproductive and respiratory syndrome virus nucleocapsid protein. J. Virol. 76, 10569–10576. 10.1128/JVI.76.20.10569-10576.2002 12239338 PMC136587

[B132] Wootton S. K. Yoo D. (2003). Homo-oligomerization of the porcine reproductive and respiratory syndrome virus nucleocapsid protein and the role of disulfide linkages. J. Virol. 77, 4546–4557. 10.1128/JVI.77.8.4546-4557.2003 12663761 PMC152152

[B133] Wysocki M. Chen H. Steibel J. P. Kuhar D. Petry D. Bates J.. (2052). Identifying putative candidate genes and pathways involved in immune responses to porcine reproductive and respiratory syndrome virus (PRRSV) infection. Anim. Genet. 43, 328–332. 10.1111/j.1365-2052.2011.02251.x 22486506

[B134] Ye M. Sun N. Li Q. Shi W. Wang B. Wang W.. (2022). Establishment and preliminary application of ELISA method of PRRSV antibody by using synthetic peptides as antigen. Chin. J. Anim. Infect. Dis. 44, 1025–1033. 10.19958/j.cnki.cn31-2031/s.20220530.002

[B135] Yoo D. Song C. Sun Y. Du Y. Kim O. Liu C. H.. (2010). Modulation of host cell responses and evasion strategies for porcine reproductive and respiratory syndrome virus. Virus Res. 154, 48–60. 10.1016/j.virusres.2010.07.019 20655963 PMC7114477

[B136] Yoo D. Wootton S. (2001). Homotypic interactions of the nucleocapsid protein of porcine reproductive and respiratory syndrome virus (PRRSV). Adv. Exp. Med. Biol. 494, 627–632. 10.1007/978-1-4615-1325-4_93 11774536

[B137] Yoo D. Wootton S. K. Li G. Song C. Rowland R. R. (2003). Colocalization and interaction of the porcine arterivirus nucleocapsid protein with the small nucleolar RNA-associated protein fibrillarin. J. Virol. 77, 12173–12183. 10.1128/JVI.77.22.12173-12183.2003 14581554 PMC254285

[B138] You J. H. Howell G. Pattnaik A. K. Osorio F. A. Hiscox A. J. (2008). A model for the dynamic nuclear/nucleolar/cytoplasmic trafficking of the porcine reproductive and respiratory syndrome virus (PRRSV) nucleocapsid protein based on live cell imaging. Virology 378, 34–47. 10.1016/j.virol.2008.04.037 18550142 PMC7103367

[B139] You X. Lei Y. Zhang P. Xu D. Ahmed Z. Yang Y.. (2022). Role of transcription factors in porcine reproductive and respiratory syndrome virus infection: a review. Front. Microbiol. 13, 924004. 10.3389/fmicb.2022.924004 35928151 PMC9344050

[B140] Yu D. Han Z. Xu J. Shao Y. Li H. Kong X.. (2010). A novel B-cell epitope of avian infectious bronchitis virus N protein. Viral Immunol. 23, 189–199. 10.1089/vim.2009.0094 20373999

[B141] Yu F. Liu L. Tian X. Chen L. Huang X. Sun Y.. (2022). Genomic analysis of porcine reproductive and respiratory syndrome virus 1 revealed extensive recombination and potential introduction events in China. Vet Sci. 9:9. 10.3390/vetsci9090450 36136666 PMC9505194

[B142] Yu F. Yan Y. Shi M. Liu H. Z. Zhang H. L. Yang Y. B.. (2020). Phylogenetics, Genomic Recombination, and NSP2 Polymorphic Patterns of Porcine Reproductive and Respiratory Syndrome Virus in China and the United States in 2014-2018. J. Virol. 94:6. 10.1128/JVI.01813-19 31896589 PMC7158704

[B143] Yu J. Liu Y. Zhang Y. Zhu X. Ren S. Guo L.. (2017). The integrity of PRRSV nucleocapsid protein is necessary for up-regulation of optimal interleukin-10 through NF-κB and p38 MAPK pathways in porcine alveolar macrophages. Microb. Pathog. 109, 319–324. 10.1016/j.micpath.2017.04.036 28457899

[B144] Yu P. Wei R. Dong W. Zhu Z. Zhang X. Chen Y.. (2019). CD163(ΔSRCR5) MARC-145 cells resist PRRSV-2 infection via inhibiting virus uncoating, which requires the interaction of CD163 with calpain 1. Front. Microbiol. 10, 3115. 10.3389/fmicb.2019.03115 32038556 PMC6990145

[B145] Zhang A. Sun Y. Jing H. Liu J. Duan E. Ke W.. (2022). Interaction of HnRNP F with the guanine-rich segments in viral antigenomic RNA enhances porcine reproductive and respiratory syndrome virus-2 replication. Virol. J. 19, 82. 10.1186/s12985-022-01811-4 35570267 PMC9107676

[B146] Zhang G. Li N. Chen Y. Zhou J. Liu H. Qi Y.. (2019). Identification of the B-cell epitopes on N protein of type 2 porcine reproductive and respiratory syndrome virus, using monoclonal antibodies. Int. J. Biol. Macromol. 130, 300–306. 10.1016/j.ijbiomac.2019.02.140 30811967

[B147] Zhang L. Li R. Geng R. Wang L. Chen X. X. Qiao S.. (2022). Tumor Susceptibility Gene 101 (TSG101) Contributes to virion formation of porcine reproductive and respiratory syndrome virus via interaction with the nucleocapsid (N) protein along with the early secretory pathway. J. Virol. 96, e0000522. 10.1128/jvi.00005-22 35080428 PMC8941886

[B148] Zhang Q. Yang F. Gao J. Zhang W. Xu X. (2022). Development of multiplex TaqMan qPCR for simultaneous detection and differentiation of eight common swine viral and bacterial pathogens. Braz. J. Microbiol. 53, 359–368. 10.1007/s42770-021-00633-w 34709596 PMC8882746

[B149] Zhao K. Li L. W. Jiang Y. F. Gao F. Zhang Y. J. Zhao W. Y.. (2019). Nucleocapsid protein of porcine reproductive and respiratory syndrome virus antagonizes the antiviral activity of TRIM25 by interfering with TRIM25-mediated RIG-I ubiquitination. Vet. Microbiol. 233, 140–146. 10.1016/j.vetmic.2019.05.003 31176400 PMC7117424

[B150] Zhao K. Li L. W. Zhang Y. J. Jiang Y. F. Gao F. Li G. X.. (2018). MOV10 inhibits replication of porcine reproductive and respiratory syndrome virus by retaining viral nucleocapsid protein in the cytoplasm of Marc-145 cells. Biochem. Biophys. Res. Commun. 504, 157–163. 10.1016/j.bbrc.2018.08.148 30172377

[B151] Zhao K. Ye C. Chang X. B. Jiang C. G. Wang S. J. Cai X. H.. (2015). Importation and recombination are responsible for the latest emergence of highly pathogenic porcine reproductive and respiratory syndrome virus in China. J. Virol. 89, 10712–10716. 10.1128/JVI.01446-15 26246582 PMC4580157

[B152] Zhao P. Jing H. Dong W. Duan E. Ke W. Tao R.. (2022). TRIM26-mediated degradation of nucleocapsid protein limits porcine reproductive and respiratory syndrome virus-2 infection. Virus Res. 311, 198690. 10.1016/j.virusres.2022.198690 35077707

[B153] Zhou L. Wang Z. Ding Y. Ge X. Guo X. Yang H.. (2015). NADC30-like strain of porcine reproductive and respiratory syndrome virus, China. Emerging Infect. Dis. 21, 2256–2257. 10.3201/eid2112.150360 26584305 PMC4672414

[B154] Zhou Y. J. An T. Q. Liu J. X. Qiu H. J. Wang Y. F. Tong Z. G.. (2006). Identification of a conserved epitope cluster in the N protein of porcine reproductive and respiratory syndrome virus. Viral Immunol. 19, 383–390. 10.1089/vim.2006.19.383 16987058

[B155] Zhu M. Li X. Sun R. Shi P. Cao A. Zhang L.. (2021). The C/EBPβ-dependent induction of TFDP2 facilitates porcine reproductive and respiratory syndrome virus proliferation. Virol. Sin. 36, 1341–1351. 10.1007/s12250-021-00403-w 34138404 PMC8209777

